# IL-21 enhances influenza vaccine responses in aged macaques with suppressed SIV infection

**DOI:** 10.1172/jci.insight.150888

**Published:** 2021-10-22

**Authors:** Daniel Kvistad, Suresh Pallikkuth, Tirupataiah Sirupangi, Rajendra Pahwa, Alexander Kizhner, Constantinos Petrovas, Francois Villinger, Savita Pahwa

**Affiliations:** 1 Department of Microbiology and Immunology, University of Miami School of Medicine, Miami, Florida, USA.; 2 Department of Biology, University of Louisiana at Lafayette, Lafayette, Louisiana, USA.; 3 Tissue Analysis Core, Immunology Laboratory, Vaccine Research Center, NIAID, NIH, Bethesda, Maryland, USA.; 4 Department of Laboratory Medicine and Pathology, Institute of Pathology, Lausanne University Hospital and Lausanne University, Lausanne, Switzerland.

**Keywords:** AIDS/HIV, Vaccines, Adaptive immunity, Immunotherapy, Influenza

## Abstract

Natural aging and HIV infection are associated with chronic low-grade systemic inflammation, immune senescence, and impaired antibody responses to vaccines such as the influenza (flu) vaccine. We investigated the role of IL-21, a CD4^+^ T follicular helper cell (Tfh) regulator, on flu vaccine antibody response in nonhuman primates (NHPs) in the context of age and controlled SIV mac239 infection. Three doses of the flu vaccine with or without IL-21–IgFc were administered at 3-month intervals in aged SIV^+^ NHPs following virus suppression with antiretroviral therapy. IL-21–treated animals demonstrated higher day 14–postboost antibody responses, which associated with expanded CD4^+^ T central memory cells and peripheral Tfh–expressing (pTfh–expressing) T cell immunoreceptor with Ig and ITIM domains (TIGIT), expanded activated memory B cells, and contracted CD11b^+^ monocytes. Draining lymph node (LN) tissue from IL-21–treated animals revealed direct association between LN follicle Tfh density and frequency of circulating TIGIT^+^ pTfh cells. We conclude that IL-21 enhances flu vaccine–induced antibody responses in SIV^+^ aged rhesus macaques (RMs), acting as an adjuvant modulating LN germinal center activity. A strategy to supplement IL-21 in aging could be a valuable addition in the toolbox for improving vaccine responses in an aging HIV^+^ population.

## Introduction

Impaired immunity in advancing age has been widely described (1–6) and is characterized by an increased susceptibility to infection and compromised efficacy of vaccination to generate antibody responses (7). Human studies have extensively demonstrated lower immunity against influenza vaccination in aged persons (8–10), with HIV-infected persons of advanced age having even greater immune deficiencies than those that are observed in natural aging (11–13). Low levels of persistent chronic inflammation, termed inflammaging (1), is a hallmark of normal aging (14) and is widely observed in virally suppressed chronic HIV–infected persons on antiretroviral therapy (ART) (15). Chronic inflammation is hypothesized to drive T cell senescence (15–17), increasing susceptibility to infection and further contributing to persistent inflammation (15). The impact of persistent chronic inflammation in chronic ART–experienced virally suppressed HIV^+^ individuals was recently highlighted by our observation that higher prevaccination frequencies of CD11b-expressing inflammatory monocytes in HIV-infected individuals correlates with poor antibody response to seasonal influenza vaccination (18). We and others have previously demonstrated impaired immune responses to influenza vaccination in HIV-infected postmenopausal women compared with HIV-uninfected age-matched controls (12), in addition to HIV-infected persons of varying ages (19, 20). Thus, strategies to improve vaccine-induced antibody responses and ameliorate chronic inflammation in aged HIV-infected persons must be explored.

Within lymph node (LN) follicles, the induction site of antigen-specific B cell responses reside in specialized CD4^+^ T cells known as T follicular helper (Tfh) cells, defined by their expression of lymphoid homing receptor CXC chemokine receptor 5 (CXCR5), whose function is to provide critical help to antigen-primed B cells for proliferation and differentiation (21). The circulating CXCR5^+^ Tfh, known as peripheral Tfh (pTfh) cells, have gained significant attention in the vaccine immunology research field (22–24). Studies in healthy adults have documented the importance of pTfh cells in the context of immune response to vaccination, as well as to various infectious diseases (13, 21). We and others have demonstrated that compromised influenza vaccine responses in both aging and HIV-infection can be attributed to lower frequencies of antigen-specific pTfh cells, along with a deficiency in IL-21 production by antigen-specific pTfh cells after vaccination (25). Additionally, In HIV-infected postmenopausal women, we reported a TNF-α–mediated impairment of CD4 and pTfh function associated with poor antibody responses to influenza vaccination (13). Interestingly, Godefroy et. al. have recently identified a subset of human pTfh cells expressing TIGIT, which exhibit strong B cell–help functions, promoting differentiation of plasmablasts and IgG production (26). While much is known about the critical role of pTfh and B cells in vaccine responses and their observed quantitative and qualitative impairment in aging and HIV infection, little is known of successful strategies to enhance or restore their function.

IL-21 is a pleiotropic γ_c_-chain signaling cytokine vital for the generation and function of Tfh cells and for LN germinal center (GC) formation in response to T-dependent and -independent antigens (21, 27). We and others have described that increased IL-21 production by pTfh cells after vaccination is associated with the magnitude of vaccine responses and induction of vaccine-specific B cell memory (25, 28). Recent studies in the cancer biology field have demonstrated that antigen-presenting cells modified to produce IL-21 potently stimulate antitumor T cell immunity in addition to avoiding IL-6/IL-17–driven inflammation (29). Biological activities of IL-21 include the promotion of B cell function/maturation and plasma cell differentiation in human B cells in conjunction with induction of CD40L on T cells (30). Our group has demonstrated the immunomodulatory potential of IL-21–IgFc therapy in acutely SIV-infected rhesus macaques (RMs) resulting in preservation of intestinal Th17 cells with lower levels of intestinal T cell proliferation, microbial translocation, and systemic inflammation/activation during chronic infection (31). We have also demonstrated that elevated plasma IL-21, IL-21R on memory B cells, and induction of functional memory B cells/plasmablasts positively correlate with vaccine titers to H1N1/09 influenza A immunization in humans (32). The data strongly suggest a critical role for IL-21 in the generation of vaccine-specific immune responses in addition to evidence for a decrease in systemic inflammation. Hence, the exploration of IL-21 as a candidate immunomodulatory agent to ameliorate chronic inflammation and improve influenza vaccine responses in aging and HIV/SIV infection is warranted.

Nonhuman primates (NHPs) have been used extensively in HIV/SIV vaccine development (33) and in the study of immune aging (34–36). In this study, we investigated the impact of IL-21 immunotherapy on antibody response to influenza vaccination in virally suppressed SIV-infected RMs on ART. The insights gained by this study provide evidence for enhanced influenza vaccine responses with IL-21 immunotherapy in old (average: 21 years, range: 3.8 years) SIV^+^ ART controlled RMs and potential mechanisms contributing to this effect.

## Results

### Age-associated immune alterations occur in ART-treated SIV-infected RM.

First, we aimed to characterize the immunologic profiles of young (average: 4.58 years, range: 6 years) and old RMs (Table 1) experimentally infected with SIV and treated with ART for the duration of the study. Average peak viral load among all animals was 1.69 × 10^5^ copies/mL (37). ART (PMPA/FTC/L-000870812) was initiated in the early chronic phase of SIV infection (day 84 after infection), and plasma viremia was suppressed to below detectable levels (<100 copies/mL) for the entire duration of the study (Supplemental Figure 1A; supplemental material available online with this article; https://doi.org/10.1172/jci.insight.150888DS1). To assess the impact of SIV infection within gut mucosal tissue, rectal biopsies were analyzed for frequencies of CD4^+^ and CD8^+^ T cells throughout acute phase of infection and following ART initiation. Following acute SIV infection, frequencies of mucosal CD4^+^ T cells declined, while frequencies of mucosal CD8^+^ T cell increased, in both young and old animals (Supplemental Figure 1, B and C). After ART initiation, frequencies of CD4^+^ T cells increased with a concomitant decrease in CD8^+^ T cells in both young and old animals. No significant differences in total CD4^+^ or CD8^+^ T cell frequencies were observed between young and old animals at any time point after ART (Supplemental Figure 1, B and C). We then analyzed circulating CD4, CD8, and respective memory subset frequencies. As anticipated, young RMs showed significantly higher baseline frequencies of total CD4 (*P* = 0.0047) (Supplemental Figure 1D), and they showed a trend of higher CD4 and CD8 naive (CD28^+^CD95^–^) subsets compared with old RMs (Supplemental Figure 1, E and I). Old RMs showed a trend for higher baseline frequencies of CD4^+^ central memory T cells (Tcm) (CD28^+^CD95^+^) (Supplemental Figure 1F), total CD8^+^ T cells (Supplemental Figure 1H), and significantly higher CD8 effector memory T cells (Tem) (CD28^–^CD95^+^) (*P* = 0.0096) subsets (Supplemental Figure 1K). No significant baseline differences in CD4^+^ Tem or CD8^+^ Tcm were observed (Supplemental Figure 1, G and J). Upon SIV infection, young animals showed a trend of declining circulating CD4^+^ T cell and increasing CD8^+^ T cell frequencies (Supplemental Figure 1, D and H). From baseline to day 42 after infection, old animals showed a significant decline in circulating CD4^+^ T cells (*P* = 0.0157) alongside a significant expansion of CD8^+^ T cell frequencies (*P* = 0.0081) (Supplemental Figure 1, D and H). Circulating CD4 naive T cell subsets expanded slightly in young RMs but remained relatively stable in old RMs before ART (Supplemental Figure 1E). CD4^+^ Tcm declined in young animals before ART, followed by a trend of expansion after ART (Supplemental Figure 1F). CD8 naive T cell subsets showed a significant expansion after ART in old RMs (*P* = 0.0383) and a trend of expansion in young RM, with young RMs showing significantly higher CD8 naive frequencies after ART (*P* = 0.0090) (Supplemental Figure 1I). Peripheral B cell subsets were also investigated, but no statistical differences were observed between young and old animals before and after SIV infection and ART initiation (Supplemental Figure 2). These results demonstrate ART-induced virologic control and partial immune reconstitution after ART initiation, with circulating and mucosal T cells of young and old RMs reflecting the immunopathology observed between young and old humans throughout viremic and ART-controlled HIV infection.

### IL-21 immunotherapy modulates influenza vaccine responses in aged SIV^+^ RMs.

**Figure 1:**
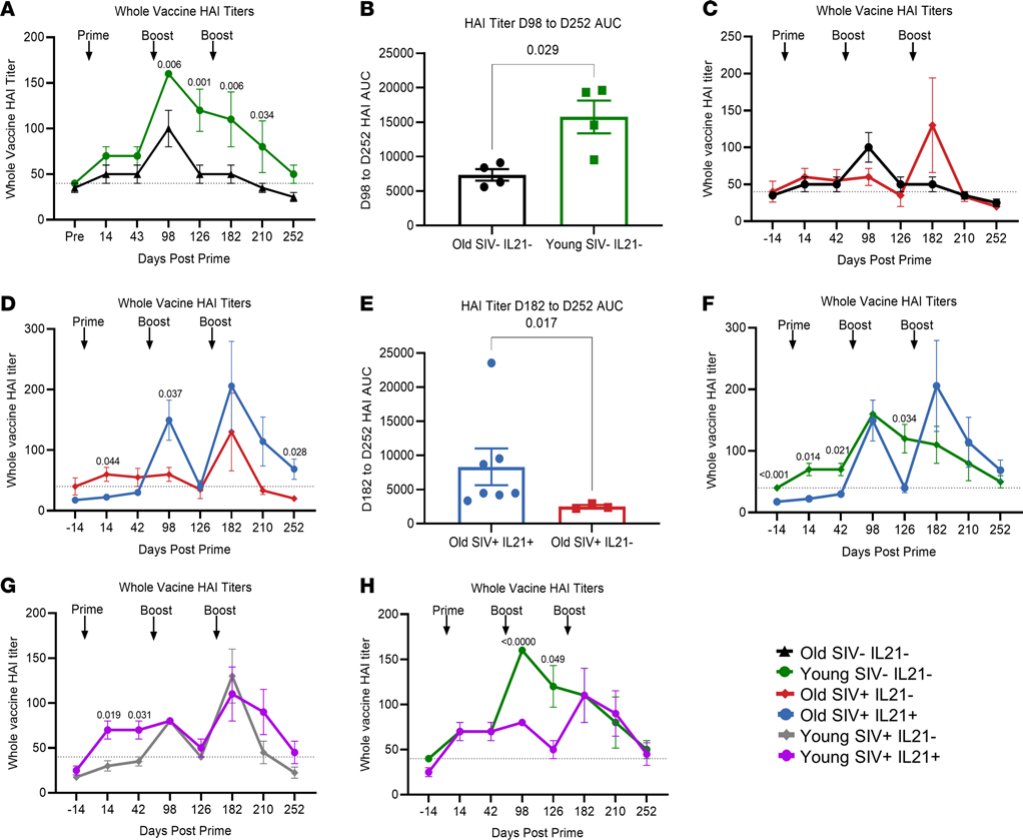
Influenza HAI titers. (**A**) Longitudinal serum titers for the whole influenza 2015–2016 vaccine as determined by HAI assay for healthy young (*n* = 4) and old (*n* = 4) control animals. (**B**) Whole vaccine HAI titer area under the curve (AUC) measured from day 98 (day 14 after B1) to day 252 (day 84 after B2) in old SIV^–^IL-21^–^ (*n* = 4) compared with young SIV^–^IL-21^–^ (*n* = 4) animals. (**C**) Longitudinal whole vaccine HAI titers of old SIV^–^IL-21^–^ (*n* = 4) compared with old SIV^+^IL-21^–^ (*n* = 4) animals. (**D**) Longitudinal whole vaccine HAI titers of old SIV^+^ IL-21^–^ (*n* = 4) compared with old SIV^+^IL-21^+^ (*n* = 8) animals. (**E**) HAI titer AUC measured from day 182 (day 14 after B2) to day 252 (day 42 after B2) in old SIV^+^IL-21^–^ (*n* = 4) compared with old SIV^+^IL-21^+^ (*n* = 8) animals. (**F**) Longitudinal whole vaccine HAI titers of young healthy (*n* = 4) compared with old SIV^+^IL-21^+^. (**G**) Longitudinal whole vaccine HAI titers of young SIV^+^IL-21^–^ (*n* = 4) compared with young SIV^+^IL-21^+^ (*n* = 4). (**H**) Longitudinal whole vaccine HAI titers of young healthy (*n* = 4) compared with young SIV^+^IL21^+^ (*n* = 4). Data are displayed as mean ± SEM. HAI titer data were analyzed by mixed-effects models, with Fisher’s LSD post hoc multiple-comparison correction and 2-tailed Mann Whitney *U* tests performed for HAI titer AUC comparisons.

Next, we measured serum influenza hemagglutination inhibition (HAI) titers in young and old SIV^–^ animals at baseline and throughout the influenza prime/boost/boost immunization schedule (Supplemental Figure 3). The rationale for this vaccination regimen of primary vaccination followed by 2 boosts was to determine whether IL-21 given at the time of boosting would bring immune responses among SIV-infected RMs to levels comparable with SIV-uninfected animals. Vaccine doses were given at 3-month intervals to optimize the development of immunologic memory. We observed significantly higher day 98 (day 14 after boost 1 [B1]) peak HAI titers (*P* = 0.006) in healthy young SIV^–^ animals compared with healthy old SIV^–^ (Figure 1A). Additionally, there was a slower postprime and postboost decline of HAI titers, as illustrated by significantly higher AUC from day 98 (day 14 after B1) to day 252 (day 84 after B2) (*P* = 0.029) in healthy young compared with healthy old animals (Figure 1B). Lower peak HAI titers observed in aged SIV^–^ animals (Figure 1A) on day 14 after B1 was further impacted by SIV^+^ infection (Figure 1C). Moreover, aged SIV^+^ animals needed 3 boosts to develop titers equivalent to aged SIV^–^. Altogether, these data indicate an age-associated impairment in immune response to flu vaccination in NHPs, highlighting the model’s efficacy in reflecting age-associated immune impairment observed in aged humans.

To investigate the immunomodulatory potential of IL-21 aimed at improving influenza vaccine responses in aged SIV^+^ RMs, we compared serum influenza HAI titers among aged SIV^+^ IL-21–untreated (*n* = 4) and aged SIV^+^ IL-21–treated (*n* = 8) RMs (Figure 1D). Aged SIV^+^ IL-21–treated animals showed significantly higher (*P* = 0.037) HAI titers on day 14 after B1 compared with aged SIV^+^ IL-21–untreated animals (Figure 1D). Although day 182 (day 14 after B2) HAI titers do not significantly differ between aged SIV^+^ IL-21–treated animals and aged SIV^+^ IL-21–untreated, day 252 (day 84 after B2) HAI titers were significantly higher among aged SIV^+^ IL-21–treated animals (*P* = 0.028) (Figure 1D). Additionally, AUCs measured from day 14 after B2 and day 84 after B2 were a significantly higher level (*P* = 0.017) in aged SIV^+^ IL-21–treated compared with untreated animals (Figure 1E), indicating a more durable antibody response among aged SIV^+^ IL-21–treated animals. Comparing influenza HAI titers among SIV^+^ IL-21–treated RMs to young SIV^–^ animals revealed no significant day 14 after B1 differences (Figure 1F), suggesting that IL-21 treatment improves post-B1 influenza HAI titers in aged SIV^+^ animals to levels comparable with SIV^–^ healthy young controls. Importantly, addition of IL-21 brought HAI titers of aged SIV^+^ to the observed peak in aged SIV^–^ levels with just 2 vaccine doses (Figure 1D) instead of the 3 doses required in non–IL-21–treated aged SIV^+^ animals to achieve comparable HAI titers (Figure 1C).

To investigate the effect of IL-21 in young animals, we compared HAI titers among young SIV^+^ IL-21–treated and young SIV^+^ IL-21–untreated animals and observed significantly higher day 14 (*P* = 0.019) and day 42 (*P* = 0.031) postprime titers; however, no differences after B1 or after B2 were observed (Figure 1G). We next compared aged SIV^+^ IL-21–treated animals with young healthy controls and observed that young healthy controls had significantly higher day 14 (*P* < 0.000) and day 42 (*P* = 0.049) post-B1 HAI titers; however, post-B2 titers did not differ from young SIV^+^ IL-21–treated animals (Figure 1H). Similar to our observations in aged SIV^+^ and SIV^–^ animals (Figure 1, C and D), young SIV^+^ animals required 3 vaccine doses to reach titers comparable with young SIV^–^ animals (Figure 1H). Unexpectedly, in contrast to aged SIV^+^ animals, the addition of IL-21 treatment in young SIV^+^ did not reduce the requirement for a third vaccine dose to achieve titers equivalent to young SIV^–^ (Figure 1, G and H), supporting the concept of impaired Tfh in the aging immune response. Taken together, these data demonstrate that IL-21 treatment appears to improve influenza vaccine–induced antibody responses in aged SIV^+^ animals but not in young SIV^+^ animals.

### Characterization of Tfh and B cells in draining LN GC after B1.

**Figure 2:**
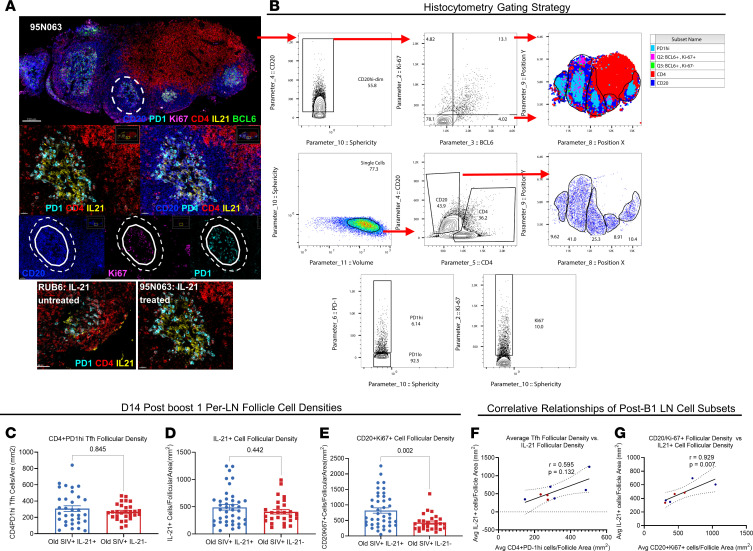
Multiplexed confocal imaging and histocytometry strategy for the comparative analysis of LN GC Tfh and B cells. (**A**) Confocal image (original magnification, ×40) showing the day 14–post-B1 draining LN and GCs from 1 old SIV^+^IL-21–treated NHP (95N063). Scale bar: 150 μm. A zoomed-in follicle (scale bar: 20–30 μm) from animal 95N063 is shown (CD20, blue; Ki-67, magenta; BCL6, green; CD4, red; PD1, cyan; and IL-21, yellow). The borders of a follicle (dotted white line) and GC (solid white line) are indicated. Representative figures showing a follicle from IL-21–untreated animal RUB6 and IL-21–treated animal 95N063 are shown. (**B**) A confocal image (original magnification, ×40) of zoomed-in follicles of 1 old SIV^+^IL-21–treated NHP (95N063) converted to histocytometry. Dotted white lines define the borders of individual follicles. The gating scheme for analysis of relevant cell populations is shown. Single cells were identified by volume and sphericity. Follicular areas were identified as CD20^hi/dim^. Analysis was performed using IMARIS and FlowJo v10. (**C**–**E**) Day 14 after B1 raw data of LN tissue Tfh, IL-21^+^, and CD20^+^Ki-67^+^ cell densities per mm^2^ for individual follicles. (**F**) Correlation between day 14 after B1 average Tfh LN densities and total IL-21^+^ LN cell densities. (**G**) Correlation between day 14 after B1 average CD20^+^Ki-67^+^ LN cell densities and total IL-21^+^ LN cell densities. All LN cell densities are per mm^2^. Due to sample quality and availability, analysis of draining LN tissue was performed with 5 of 8 animals from old SIV^+^IL-21^+^ and 3 of 4 animals from old SIV^+^IL-21^–^ groups. Data are displayed as mean ± SEM, with 2-tailed Mann Whitney *U* tests, and Spearman’s R correlations performed.

To further characterize the influenza vaccine–induced responses at the inductive site of the immune response, we asked whether LN follicle GC reactions were altered in IL-21–treated animals on day 14 after B1, the time point at which we observed improved vaccine responses among IL-21–treated animals (Figure 1E). Quantitative, multiplexed confocal imaging for histocytometric analysis of follicular Tfh and B cell populations was performed using tissues obtained from draining LNs at day 14 after B1 (38) (Figure 2, A and B). Despite the limited access to LNs, we observed that Tfh cell density (CD4^+^PD-1^hi^/mm^2^) per follicle was not significantly different between IL-21–treated and –untreated animals (Figure 2C). In addition to follicular Tfh density, we measured the density of follicular CD20^+^Ki-67^+^ B cells and total follicular IL-21^+^ cells in post-B1 draining LN tissues. Per-follicle IL-21^+^ cell density did not statistically differ between groups (Figure 2D); however, per-follicle CD20^+^Ki-67^+^ GC B cell densities were significantly higher (*P* = 0.0016) in IL-21–treated animals compared with IL-21–untreated (Figure 2E). Average per-animal follicular Tfh density did not significantly associate (*r* = 0.5952, *P* = 0.132) with average IL-21^+^ cell density. However, follicular CD20^+^Ki-67^+^ cell density correlated directly (*r* = 0.9286, *P* = 0.007) with average IL-21^+^ cell density (Figure 2, F and G). These data demonstrate that the density of IL-21^+^ cells within LN follicles associates with proliferating follicular B cell density after B1, pointing toward increased GC activity in IL-21–treated animals.

### IL-21–treated aged SIV^+^ RMs have expanded TIGIT^+^CD4^+^ Tcm and TIGIT^+^ pTfh cells after vaccination.

**Figure 3:**
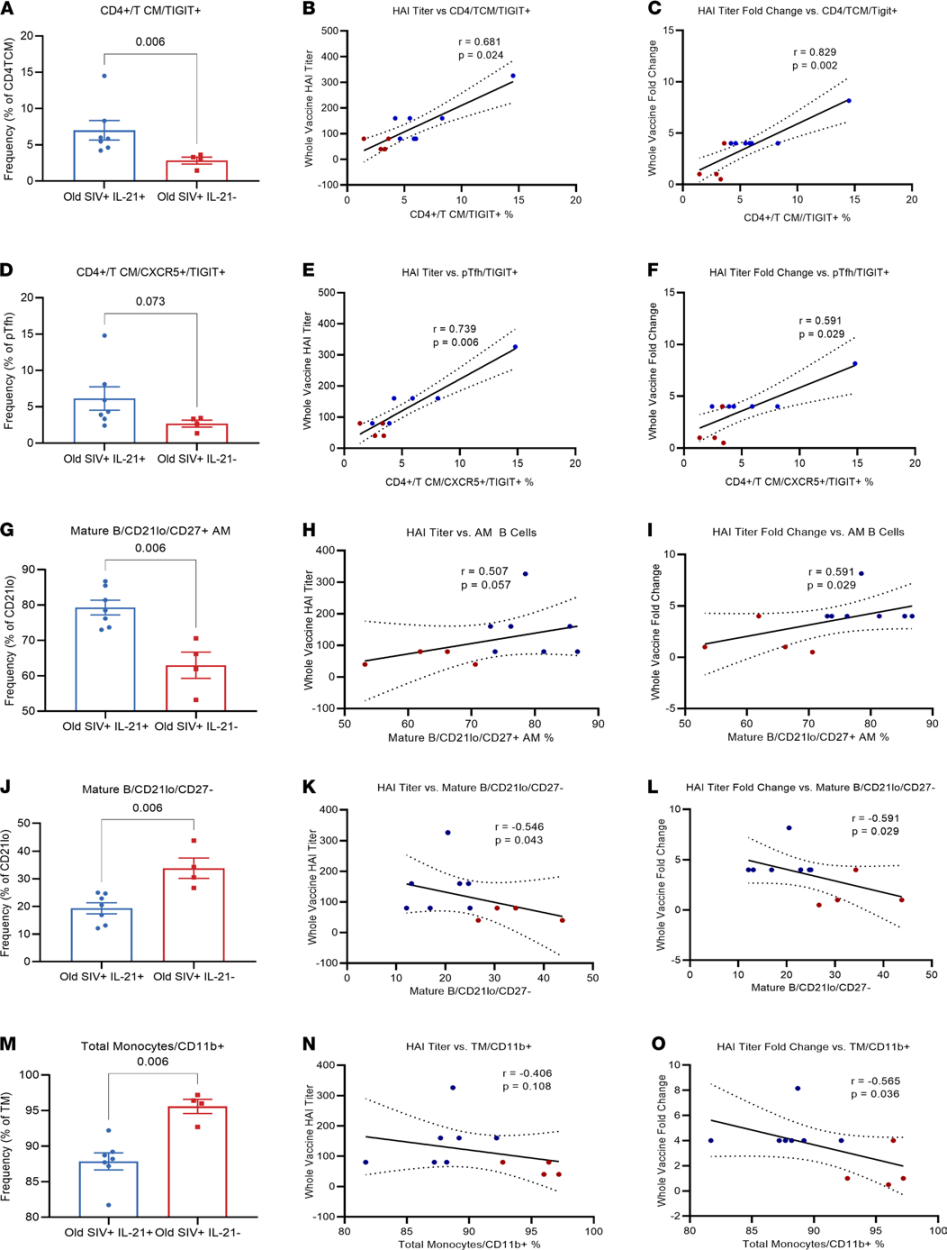
Post-B1 CD4^+^ Tcm, pTfh, B cell, and monocyte phenotypes and correlation with whole vaccine HAI titer. (**A**–**F**) Day 14–post-B1 TIGIT^+^ frequencies of CD4^+^ Tcm and pTfh (CD4^+^/Tcm/CXCR5^+^) and correlations with day 14–post-B1 whole vaccine HAI titers and HAI titer fold change from B1 baseline. (**G**–**L**) Day 14–post-B1 CD27^+^ AM B cell and CD27^–^ DN B cell frequencies of CD21^lo^ mature B cells and correlations with day 14–post-B1 whole vaccine HAI titers and HAI titer fold change from B1 baseline. (**M**–**O**) Day 14–post-B1 CD11b^+^ frequencies of total monocytes (including Lineage^–^, CD14^+^CD16^–^, CD14^+^CD16^+^, and CD14^–^CD16^+^) and correlation with day 14–post-B1 whole vaccine HAI titers and HAI titer fold change from B1 baseline. Blue dots represent old SIV^+^IL-21^+^ animals (due to missing sample from one animal, *n* =7), while red squares represent old SIV^+^IL-21^–^ animals (*n* = 4). Data are displayed as mean ± SEM, with 2-tailed Mann Whitney *U* test and Spearman’s R correlations performed.

Given the increased GC reactivity in the draining LNs, we sought to investigate if there were IL-21–induced alterations in circulating CD4 and CD8 subsets using a multiparametric flow cytometry (Supplemental Figure 4A), which may provide translationally relevant immunologic correlates of improved vaccine responses. No significant differences were found for several populations analyzed (Supplemental Figure 5, A and B). However, upon further phenotypic characterization of CD4^+^ Tcm, we observed significantly elevated (*P* = 0.0061) frequencies of TIGIT-expressing CD4^+^ Tcm day 14 after B1 (Figure 3A), the same time point at which SIV^+^ IL-21–treated animals had significantly improved influenza HAI titers compared with SIV^+^ IL-21–untreated animals (Figure 1E). Interestingly, pTfh cells expressing TIGIT have recently been shown to exhibit strong B cell help functions and high levels of IL-21 production, and they have been shown to promote differentiation of plasmablasts and IgG production (26, 39). Furthermore, we found that there was a trend (*P* = 0.0727) of increased TIGIT expression on pTfh cells in IL-21–treated animals (Figure 3D), and influenza HAI titers day 14 after B1 correlated with both TIGIT^+^CD4^+^ Tcm (*r* = 0.6808, *P* = 0.0120) (Figure 3B) and TIGIT^+^ pTfh (*r* = 0.7387, *P* = 0.0112) cells (Figure 3E). Fold change in HAI titer showed significant positive association with TIGIT^+^CD4^+^ Tcm (*r* = 0.8291, *P* = 0.0008) (Figure 3C) and TIGIT^+^ pTfh (*r* = 0.5914, *P* = 0.0285) (Figure 3F). Similar observations were made day 14 after B2, with significantly higher frequencies of TIGIT^+^CD4^+^ Tcm (*P* = 0.0476) and pTfh (*P* = 0.0190) (Supplemental Figure 6, A–C) in aged SIV^+^ IL-21–treated animals compared with aged SIV^+^ IL-21–untreated controls, which showed direct association with HAI titers and fold change in titer at day 14 after B2 (Supplemental Figure 6C). Interestingly, the frequencies of TIGIT^+^CD4^+^ Tcm and TIGIT^+^ pTfh on day 14 after B1 observed in aged SIV^+^ IL-21–treated animals were not significantly different than frequencies observed in young SIV^–^ healthy IL-21 untreated controls, and although not statistically significant, there was a similar positive association between HAI titers and the frequency of TIGIT-expressing CD4^+^ Tcm (*r* = 0.507, *P* = 0.125) and pTfh (*r* = 0.2267, *P* = 0.3) cells among healthy young and old IL-21–untreated controls (Supplemental Figure 7A). These observations demonstrate that TIGIT-expressing CD4^+^ Tcm and pTfh are increased by IL-21 administration and correlate with influenza vaccine antibody responses in aged SIV^+^ RMs.

### IL-21 treatment induces expansion of activated memory B cells associated with improved vaccine responses.

We next asked if IL-21–treated animals displayed alterations in the circulating memory B cell compartment (Supplemental Figure 4B). In data not shown, neither total circulating CD20^+^ B cells nor Lin^–^CD20^+^CD10^–^ mature B cell frequencies differed between IL-21–treated and –untreated animals. However, frequencies of Lin^–^CD20^+^CD10^–^CD21^lo^CD27^+^ activated memory (AM) B cells were significantly higher in aged SIV^+^ IL-21–treated animals at day 14 after B1 (*P* = 0.0040) compared with aged IL-21–untreated animals (Figure 3G). Moreover, frequencies of Lin^–^CD20^+^CD10^–^CD21^lo^CD27^–^ B cells (hereafter referred to as CD21^lo^CD27^–^) were significantly lower in the SIV^+^ IL-21–treated animals compared with IL-21–untreated (*P* = 0.0060) (Figure 3J) and SIV^–^ IL-21–treated animals (*P* = 0.0419) (Supplemental Figure 7B). Frequencies of AM B cells at day 14 after B1 positively correlated (*r* = 0.5914, *P* = 0.0285) with fold changes in HAI titer after B1 (Figure 3I) and showed a trend of positive association with HAI titers after B1 (Figure 3H). However, frequencies of CD21^lo^CD27^–^ B cells at day 14 after B1 showed negative association with post-B1 HAI titers (*r* = –0.546, *P* = 0.043), as well as HAI titer fold change (*r* = –0.5914, *P* = 0.0285) after B1 (Figure 3, K and L), indicating that IL-21 appears to induce alterations in memory B cell subset distribution associated with improved vaccine responses. Similarly, on day 14 after B2, old SIV^+^ IL-21–treated animals had higher frequencies of AM B cells (*P* = 0.0283) and lower frequencies of CD21^lo^CD27^–^ B cells (*P* = 0.0242) (Supplemental Figure 6C), showing trends of positive and negative association with HAI titers, respectively (Supplemental Figure 6C).

Given that CD4^+^ Tcm cells and pTfh provide critical help to B cells for differentiation and memory, we investigated if a relationship exists between AM B cells and the expanded frequencies and expression of TIGIT^+^CD4^+^ Tcm and TIGIT^+^ pTfh cells observed in IL-21–treated animals. Our data indicate that post-B1 frequencies of AM B cells correlated significantly with frequencies (*r* = 0.6091, *P* = 0.0260) and median fluorescence intensity (MFI) (*r* = 0.5421, *P* = 0.0442) of TIGIT^+^CD4^+^ Tcm (Supplemental Figure 8, A and B). Additionally, we observed that TIGIT MFI, not frequency, on pTfh cells correlated significantly (*r* = 0.6804, *P* = 0.0125) with AM B cell frequencies (Supplemental Figure 8, C and D). These data show that there is likely a relationship between vaccine responses and the concomitant expansion of AM B cells and TIGIT expression on both CD4^+^ Tcm and pTfh cells.

### IL-21 treatment lowers the frequencies of circulating CD11b^+^ inflammatory monocytes.

Next, we studied the impact of IL-21 on monocyte subset distribution (18, 40) (Supplemental Figure 4C). A subset of monocytes expressing integrin CD11b was significantly (*P* = 0.0061) lower in SIV^+^ IL-21–treated animals compared with IL-21–untreated animals (Figure 3M), and levels of CD11b monocytes showed a negative association (*r* = 0.5650, *P* = 0.0364) with post-B1 HAI titer fold changes (Figure 3O) and nonsignificant negative association with post-B1 HAI titers (Figure 3N). Moreover, frequencies of CD11b-expressing monocytes correlated negatively with frequencies of TIGIT^+^CD4^+^ Tcm (*r* = –0.6545, *P* = 0.0168) (Supplemental Figure 8E), MFI of TIGIT on CD4^+^ Tcm (*r* = –0.7927, *P* = 0.0025) (Supplemental Figure 8F), and TIGIT MFI on pTfh cells (*r* = –0.7900, *P* = 0.0027) (Supplemental Figure 8G). Additionally, post-B1 frequencies of CD11b^+^ monocytes in SIV^+^ IL-21–treated animals were significantly lower than both old healthy IL-21–untreated controls (*P* = 0.0010) and young healthy IL-21–untreated controls (*P* = 0.0277) (Supplemental Figure 7C).

Altogether, these data suggest that the observed enhancement of antibody responses to flu vaccination in the IL-21–treated animals was associated with IL-21–induced alterations in immune cell compartments, including increased frequencies of TIGIT^+^CD4^+^ Tcm and TIGIT^+^ pTfh cells; the concomitant expansion of AM B cells and contraction of CD21^lo^CD27^–^ B cells; and reduction in frequencies of CD11b-expressing monocytes. Furthermore, prior to prime vaccination and IL-21 administration, frequencies of the aforementioned circulating immune cell populations did not differ between RM groups (Supplemental Figure 9, A–F), suggesting that IL-21 immunotherapy directly or indirectly modulates circulating CD4^+^, B cell, and monocyte subsets.

### Frequencies of TIGIT and PD-1 on CD4 and pTfh cell subsets on the day of vaccination are predictive of antibody responses.

**Figure 4:**
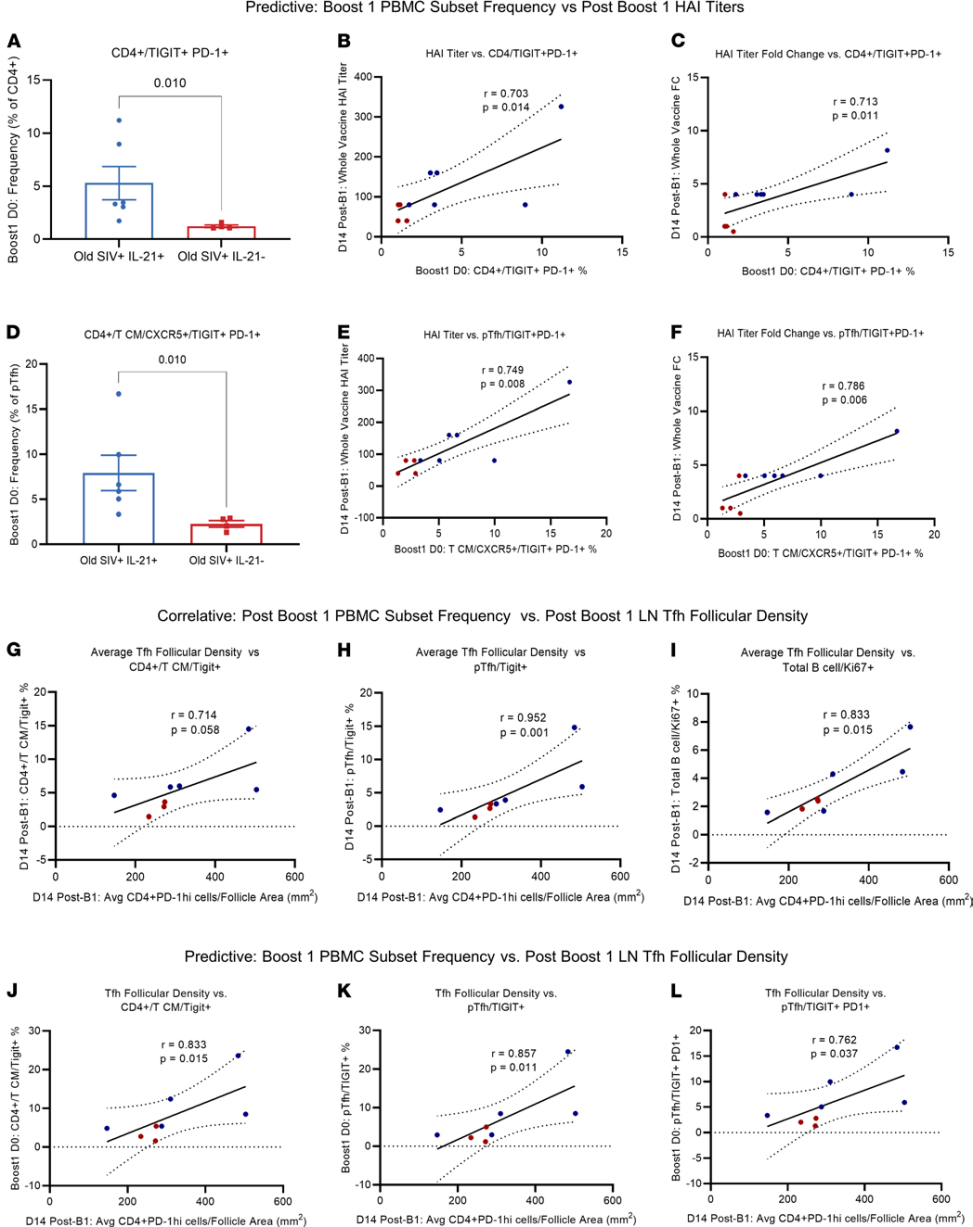
PBMC subset frequencies predict post-B1 HAI titers and LN Tfh follicular density. Correlative relationships represent variables that are measured at the same time point, while predictive relationships are between variables, measured on the day of B1 (B1, day 0), which correlate with variables measured at a later time point (day 14 after B1). (**A**–**F**) Day of B1 TIGIT^+^PD-1^+^ double-positive frequencies of total CD4^+^ and CD4^+^ Tcm and correlations with day 14–post-B1 whole vaccine HAI titers and HAI titer fold change from B1 baseline. (**G**–**I**)Spearman’s correlations between day 14–post-B1 average LN follicle Tfh (CD4^+^PD-1^hi^) density and day 14–post-B1 peripheral CD4^+^/Tcm/TIGIT^+^ frequencies, day 14–post-B1 CD4^+^/Tcm/CXCR5^+^/TIGIT^+^ frequencies, and day 14–post-B1 frequencies of Ki-67^+^ total B cells. (**J**–**L**) Predictive correlations of B1 day 0 PBMC CD4^+^/Tcm/TIGIT^+^ frequencies, B1 day 0 CD4^+^/Tcm/CXCR5^+^/TIGIT^+^ frequencies, and B1 day 0 CD4^+^/Tcm/CXCR5^+^/TIGIT^+^PD-1^+^ frequencies all correlated with day 14–post-B1 average LN follicle Tfh density per animal. Due to sample quality and availability, analysis of draining LN tissue and the relationship with peripheral subset frequencies was performed with 5 of 8 animals from old SIV^+^IL-21^+^ and 3 of 4 animals from old SIV^+^IL-21^–^ groups. All LN cell densities are per mm^2^. Data are displayed as mean ± SEM, with 2-tailed Mann Whitney *U* test and Spearman’s R correlations performed.

We then asked if day –2 IL-21 priming prior to B1 vaccination modulated circulating CD4 and pTfh subsets on the same day as B1 vaccination such that altered phenotypes may be predictive of day 14 after B1 vaccine responses. On the day of vaccination, frequencies of TIGIT and PD-1–double positive total CD4 (*P* = 0.0037) and pTfh (*P* = 0.0025) cells were significantly higher in the IL-21–treated SIV^+^ animals compared with IL-21–untreated animals (Figure 4, A and D). Additionally, HAI titer after B1, as well as fold change in HAI titers after B1, significantly correlated with the frequency of TIGIT^+^PD-1^+^CD4^+^ (*r* = 0.7031, *P* = 0.0138; *r* = 0.7128, *P* = 0.0111) and pTfh (*r* = 0.7487, *P* = 0.0077; *r* = 0.7677, *P* = 0.0056) cells at the time of vaccination, respectively (Figure 4, B, C, E, and F). These data show that IL-21 priming prior to boost vaccination may be inducing the expression of TIGIT and PD-1 on both CD4 and pTfh cell subsets, which are predictive of vaccine responses after B1.

### IL-21 modulates LN immune cell subset dynamics, which strongly associate with peripheral blood correlates of vaccine response.

We next aimed to further characterize the relationship between our findings in peripheral blood and draining LN tissue on day 14 after B1. We observed that average follicular Tfh cell density per follicle area mm^2^ was significantly correlated with the frequency of post-B1 TIGIT^+^CD4^+^ Tcm cells (*r* = 0.7143, *P* = 0.0288) and showed a direct association with the frequency of TIGIT^+^ pTfh cells after B1 (*r* = 0.9524, *P* = 0.0006) (Figure 4, G and H, and Figure 2, A and B). Furthermore, we also observed that average follicular Tfh cell density significantly correlated with total proliferating B cells expressing Ki-67 in the blood (*r* = 0.833, *P* = 0.0077) (Figure 4I). Similarly, average follicular IL-21^+^ cell density at day 14 after B2 (Supplemental Figure 10A) correlated with the frequency of TIGIT^+^ pTfh cells after B2 (*r* = 0.8857, *P* = 0.0167) (Supplemental Figure 10B), as well as average follicular Tfh density after B2 (*r* = 0.8857, *P* = 0.0167) (Supplemental Figure 10, C and D). These data indicate that the density of Tfh within LN follicles, a metric of GC reactivity, is closely associated with circulating TIGIT^+^CD4^+^ Tcm and, to a further extent, with TIGIT^+^ pTfh cells.

We next asked if day –2 IL-21 priming–induced alterations in circulating CD4^+^ Tcm and pTfh subsets at the time of B1 were predictive of GC reactivity, as measured by per-animal average follicular Tfh density on day 14 after B1. We found that the follicular Tfh density after B1 was positively correlated with frequencies of TIGIT^+^CD4^+^ Tcm (*r* = 0.8333, *P* = 0.0077), TIGIT^+^ pTfh (*r* = 0.8571, *P* = 0.0054), and TIGIT^+^PD-1^+^ pTfh (*r* = 0.7619, *P* = 0.0184) (Figure 4, J–L) at the time of boost. These findings indicate that the relative degree of GC activity as a function of follicular Tfh density can be predicted by frequencies of TIGIT^+^CD4^+^ Tcm, TIGIT^+^ pTfh, and TIGIT^+^PD-1^+^ pTfh at the time of vaccination, and that IL-21 priming prior to vaccination induces expansion of said circulating CD4^+^ T cell subsets and subsequent increased follicular Tfh density.

These data show a consistent relationship between postboost follicular Tfh cell density in draining LNs and circulating TIGIT^+^ pTfh cells, a subset that correlates with HAI titers. Taken together, our data demonstrate that vaccine responses are improved in SIV^+^ IL-21–treated older animals compared with IL-21–untreated animals, and that IL-21 appears to induce significant alterations in circulating and LN follicle immune cell populations that are correlative of vaccine-induced antibody production.

### Multivariate dimensionality reduction and discriminative variable selection analysis for data integration.

In order to better understand the IL-21–induced immunomodulatory effects at day 14 after B1, we employed a statistical data integration method using sparse partial least squares–discriminant analysis (sPLS-DA). The sPLS-DA is a supervised clustering machine-learning algorithm for multivariate dimensionality reduction, parameter selection, and classification (41). Prior to running sPLS-DA, we employed unsupervised principal component analysis (PCA) as an indicator of sPLS-DA reliability on our flow cytometry data set (41) (Supplemental Figure 11, A and B). Because PCA modeling is unsupervised, without input of group identity, a combined 53% explained variance from principal component 1 (PC1) and PC2 in addition to spatial separation of IL-21–treated versus –untreated animals when plotting PC1 against PC2 supports the reliability of sPLS-DA as a tool for both variable selection and classification in our data set (41).

**Figure 5:**
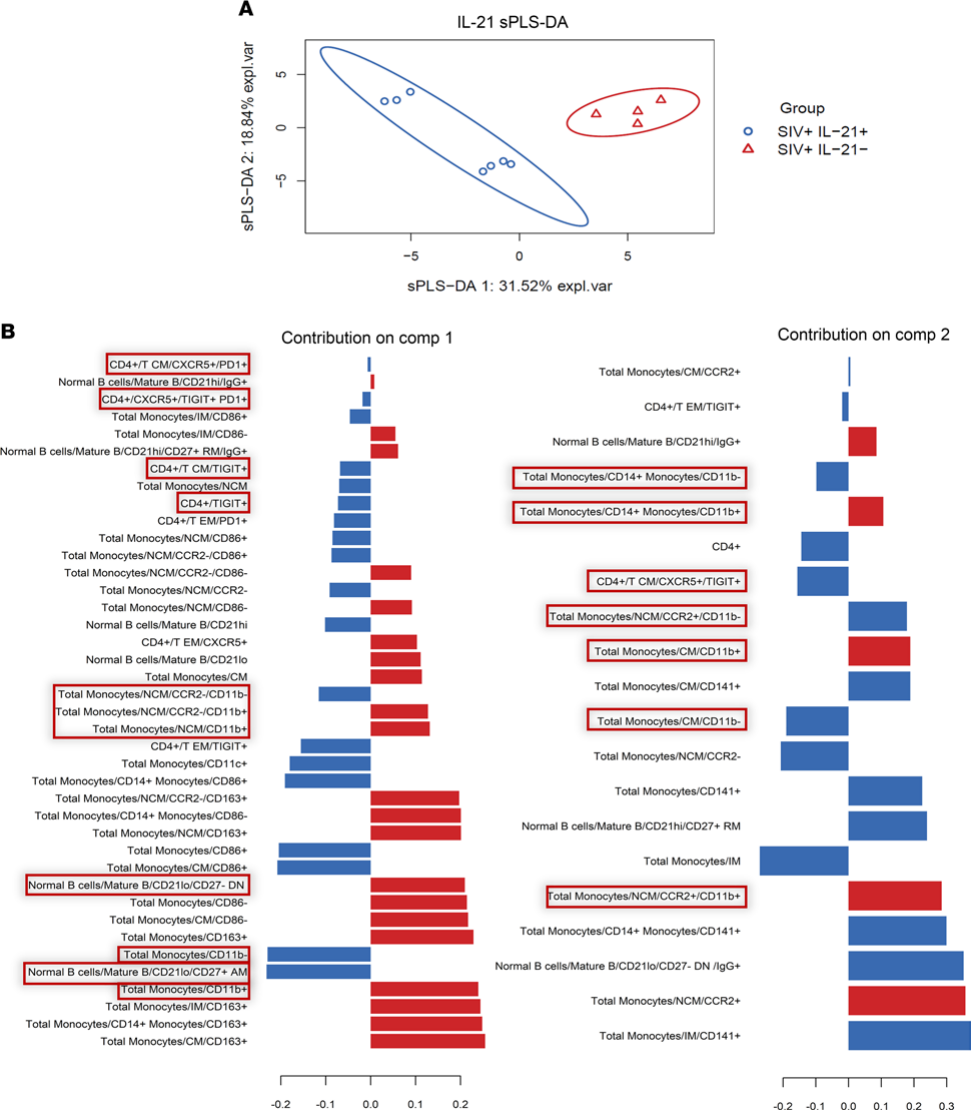
Day 14–post-B1 PBMC sparse partial least squares-discriminant analysis (sPLS-DA) modeling for variable selection and classification. (**A**) sPLS-DA components 1 and 2 plotted on the *x* and *y* axis, respectively. Dots indicate individual animals, with blue indicating IL-21–treated animals (*n* = 7) and red indicating controls (*n* = 4). (**B**) The loading variables and their loading scores are shown for sPLS-DA components 1 and 2. Peripheral blood immune subsets similar to those identified to be altered by IL-21 immunotherapy through univariate analysis are indicated by red boxes. sPLS-DA was performed using R.

The sPLS-DA modeling revealed 2 primary components accounting for a combined explained variance of 50.36 %, with PC1 and PC2 accounting for 31.52% and 18.84% of explained variance, respectively (Figure 5A). A third component was also identified; however, it was not focused on, due to low (10.49%) explained variance in addition to being composed of immunologic variables that have no significant correlation with HAI titers. In agreement with our univariate analysis, sPLS-DA modeling revealed that TIGIT^+^ pTfh, CD21^lo^CD27^–^ B cells, AM B cells — as well as CD11b^+^/CD11b^–^ monocyte populations — were among the top loadings of components 1 and 2 that differentiate IL-21–treated animals from control animals after B1 (Figure 5B). Other variables present in the top loadings of components 1 and 2 include CD163- and CD86-expressing monocytes, as well as resting memory B cell frequencies (Figure 5B). However, in data not shown, neither resting memory B cell, CD163, nor CD86 frequencies on monocyte populations had significant univariate linear relationships with HAI titers, so these subsets were not further investigated.

We next wanted to test for interaction between TIGIT^+^ pTfh, AM B cells, and CD11b^+^ total monocytes to determine if their frequencies on day 14 after B1 can predict vaccine response alone and/or together, while controlling for IL-21 immunotherapy (Table 2). To test this, we ran a Poisson regression model, employing a top-down exploratory model selection approach, and found significant main effects for each TIGIT^+^ pTfh, AM B cells, CD11b^+^ monocytes, and IL-21 immunotherapy, suggesting that these population frequencies, as well as IL-21 treatment status, can predict vaccine response after B1. Furthermore, we also identified a significant interaction between TIGIT^+^ pTfh × AM B cells and TIGIT^+^ pTfh × CD11b^+^ total monocytes. Given the Poisson regression model results for day 14 after B1, we then asked if we could predict day 14–post-B1 vaccine response with cell subset frequencies at the preprime and pre–IL-21 immunotherapy baseline time point. For this, we built a Poisson regression model to show the effects of baseline frequency of TIGIT^+^ pTfh, AM B cells, and CD11b^+^ monocytes on HAI titer level on day 14 after B1, while controlling for IL-21 immunotherapy (Table 3). This modeling revealed main effects of TIGIT^+^ pTfh, AM B cell, and IL-21 immunotherapy, as well as interaction effects of TIGIT^+^ pTfh × CD11b^+^ total monocytes, TIGIT^+^ pTfh × AM B cell, and TIGIT^+^ pTfh × AM B cell × CD11b^+^ monocytes (3-way). Taken together, the sPLS-DA and Poisson modeling improves our understanding of immunomodulatory effects of IL-21 immunotherapy in an SIV^+^ aging RM model and emphasizes the interrelatedness of TIGIT^+^ pTfh, AM B cells, and CD11b^+^ monocytes and their significant influence on flu vaccine response.

## Discussion

In the current study, we tested the hypothesis that an in vivo immunomodulatory approach using IL-21 given at the time of immunization would augment the antibody response to flu vaccination in an aging and SIV-infection/ART-treated NHP model. To test our hypothesis, we administered influenza vaccination in a prime/boost/boost strategy at 3-month intervals with and without s.c. IL-21–IgFc administration on days –2, 0, and 7 of each vaccination in the NHP model of SIV infection in aged RMs. The rationale for this study was based on several findings. First, IL-21 has been shown to promote B cell function/maturation, as well as plasma cell differentiation, in concert with CD40-CD40L signaling via Blimp1 (30). Second, in vivo administration of IL-21 has the capacity to preserve intestinal Th17 cells and maintain mucosal barrier integrity in early SIV infection, limiting microbial translocation and systemic inflammation in the chronic phase of infection (31). We have also previously demonstrated in SIV-infected RM that IL-21 did not induce T cell activation or proliferation, and it did not increase SIV viral load (42). Furthermore, in humans, our group found that increased plasma IL-21, IL-21R on memory B cells, and expansion of functional memory B cells and plasmablasts all positively associated with H1N1/09 Influenza A immunization–induced vaccine titers (32). Altogether, these studies led us to hypothesize that IL-21 adjuvanted influenza vaccination would induce potent humoral responses in aged SIV^+^ RMs. In this study, we show that IL-21 immunotherapy significantly improves influenza vaccine responses in aged SIV^+^ RMs and point to the potential of IL-21 in vaccinology for special populations.

Our aging SIV NHP model validates age-associated immune impairment characterized by altered immune cell subset distribution and lower antibody responses to flu vaccination. One limitation of the current study is the use of primarily female animals; these animals were retired from breeding (study naive) and truly aged, > 18 years old, which are rarely maintained and available at primate centers. Generally, males of this age have been enrolled in other studies and are no longer available, hence the focus on female animals. In this study, we demonstrate that IL-21 immunotherapy significantly improves influenza vaccine responses in aged SIV^+^ RMs but not young SIV^+^ RMs. We observed improved flu vaccine–induced antibody levels in the old SIV^+^ IL-21–treated animals, particularly at day 14 after B1, in which raw and fold change (from B1 baseline) HAI titers were significantly higher among IL-21–treated SIV^+^ aged animals compared with controls. Within draining LN tissues after B1, we observed significantly higher densities of proliferating Ki-67^+^ B cells, indicating improved GC reactions among IL-21–treated animals. Given the widely published evidence for the critical role of Tfh and pTfh cells in providing B cell help for the production of antibodies (25, 43), we sought to investigate the translationally relevant peripheral blood pTfh cells in further detail utilizing a flow cytometry panel designed to characterize the expression of immune checkpoint molecules PD-1 and TIGIT. These molecules are highly expressed on conventional GC Tfh cells in addition to pTfh cells; therefore, they represent markers of interest when studying pTfh (23, 43, 44).

Godefroy et al. described a unique subset of TIGIT-expressing pTfh cells, which exhibit strong B cell help functions, promoting differentiation of plasmablasts and IgG production (26). In our study, we observed elevated frequencies of TIGIT-expressing CD4^+^ Tcm and TIGIT-expressing pTfh cells in IL-21–treated animals on day 14 after B1, and this correlated positively with day 14–post-B1 vaccine–induced antibody titers, indicating that these cells may be induced by IL-21 treatment and represent immune correlates of flu vaccine responsiveness. It is interesting to note that IL-21 treatment in aged SIV^+^ animals improved day 14–post-B1 HAI titers to levels observed in young healthy controls. These findings indicate that increased postvaccination TIGIT expression on CD4^+^ Tcm and pTfh may represent an immune correlate of ongoing immune responses. TIGIT binds to its high-affinity receptor CD155, which is expressed by both memory and naive B cell subsets (45). Previous research has shown that the coculture of TIGIT-expressing pTfh with B cells improved IgG production by B cell subsets, while TIGIT-blocking antibodies decreased the response, indicating a direct role of TIGIT in B cell help (26, 45). These findings support our observation of improved antibody responses among IL-21–treated animals with a higher frequency of TIGIT-expressing pTfh after B1. Additionally, in murine T cells, IL-21 has been shown to induce the expression of the transcription factor Blimp1 through functional cooperation of STAT3 and IRF4, both of which are directly induced by IL-21 signaling (44). Furthermore, it has been shown that BLIMP1 binds to the promotors of PD-1 and TIGIT, directly upregulating their expression (46). Together, these findings represent a potential mechanism by which IL-21 immunotherapy induces TIGIT upregulation on CD4 pTfh cells in the current study.

To characterize Tfh at the induction site of the immune response, we performed in situ analysis of draining LN tissues and histocytometry analysis of immune cell densities within LN follicles. Our finding of a direct association between average follicular density of Tfh cell and frequencies of circulating TIGIT^+^CD4^+^ Tcm, and a near direct association with TIGIT^+^ pTfh cells, indicates that IL-21 immunotherapy in flu vaccination may exert its immunomodulatory effects in both LN tissue and peripheral blood compartments. We have previously described compromised GC activity in healthy aged RM compared with healthy young RM as characterized, in part, by lower follicular Tfh density (38), indicating that lower Tfh density within LN follicles is representative of impaired immunity in RM. Compromised GC activity as a function of decreased follicular Tfh density may represent intrinsic defects in the maintenance and differentiation of GC Tfh cells or dysfunctional regulation between Tfh and B cells within LN GCs (47). These data indicate that the Tfh dynamics within the inductive site of the vaccine-induced immune response on day 14 after B1 may be altered by IL-21 administration, and that Tfh density within LN follicles is closely related to the circulating frequencies of TIGIT-expressing CD4^+^ Tcm and pTfh cells. Although the density of Tfh was not directly altered in IL-21–treated animals, we did observe increased density of Ki-67^+^ B cells. This could indicate that (a) the quality of Tfh is different in the presence of IL-21 or (b) the administered IL-21 has a direct effect on B cells that bypasses or supplements Tfh-derived B cell help; in both cases, there is increased GC reactivity in aged SIV^+^ IL-21–treated animals.

Upon characterization of B cell responses after B1, we observed increased Ki-67^+^ B cell density within LN follicles of IL-21–treated old SIV^+^ animals, and it correlated strongly with follicular IL-21^+^ cell density. Among peripheral B cell subsets, we found increased levels of circulating AM B cells, as well as a concomitant decrease of CD21^lo^CD27^–^ B cells at day 14 after B1 and day 14 after B2 in IL-21–treated animals. Furthermore, the frequencies of AM and CD21^lo^CD27^–^ B cells correlated positively and negatively, respectively, with vaccine-induced antibody titers, suggesting that IL-21 treatment may alter B cell differentiation after B1, with similar trends observed after B2. Additionally, the day 14–post-B1 AM and CD21^lo^CD27^–^ B cell frequencies observed in IL-21–treated animals were comparable with those frequencies observed in young healthy controls, which indicates that IL-21 treatment in aged SIV^+^ animals may have the capacity to restore AM and CD21^lo^CD27^–^ B cell frequencies after vaccination in a manner that resembles young healthy control animals. Ligation of B cell receptor (BCR) with soluble antigen — alongside CD4-derived B cell help mediated by molecules including CD40-CD40L and ICOS-ICOSL, in addition to Tfh-derived IL-21 — induces activation of B cells to undergo maturation, class switch recombination, somatic hypermutation, and antibody production (48, 49). Memory B cells are unable to become activated in the absence of T cell help (50); thus, the observed increase in AM B cells among IL-21–treated animals compared with controls may suggest that there was improved CD4-derived help, possibly mediated by the expanded post-B1 frequencies of TIGIT-expressing CD4^+^ Tcm and pTfh previously described to provide strong B cell help — or by direct effect of IL-21 on B cells. The negative association of CD21^lo^CD27^–^ B cells with vaccine-induced antibody titers after B1 is supported by evidence that CD21^lo^CD27^–^ B cells have been shown to associate with immune senescence, exhaustion, and autoimmunity; in HIV-infected humans, their frequencies are expanded compared with healthy controls and inversely correlate with levels of plasmablasts following influenza immunization (20, 51).

IL-21 treatment in aged SIV^+^ RMs reduces frequencies of CD11b-expressing monocytes, which negatively correlate with HAI titers. We studied the impact of IL-21 on monocyte subset distribution in light of recent reports linking CD11b^hi^-expressing monocytes with higher levels of inflammatory cytokine secretion, lower CD4^+^ T cell proliferation, diminished postvaccination influenza antibody titers, greater arterial thickness, and elevated plasma levels of CRP, LPS, and sCD14 in HIV-infected persons (18, 40, 52). Upon characterization of circulating monocytes, we observed lower frequencies of CD11b-expressing total monocytes in IL-21–treated RM on day 14 after B1 compared with controls. Interestingly, our group has previously shown in humans that CD11b^hi^-expressing monocytes are highly proinflammatory (production of TNF-α and IL-6 following LPS stimulation) compared with CD11b^lo^, and they negatively associate with influenza vaccine–induced antibody titers in HIV^+^ individuals (18). Additionally, our group and others have described inhibitory effects of monocytes on antigen-specific T cell proliferation (18, 53). Specifically, we previously showed that CD11b^hi^ inflammatory monocytes cocultured with antigen-specific CD4^+^ T cells resulted in reduced T cell proliferation (18). Furthermore, in mice, it has been shown that inflammatory monocytes have the capacity to migrate to LNs and impair T cell responses to vaccination (53). The decreased frequencies of CD11b-expressing monocytes observed in IL-21–treated RM may be indicative of lower systemic inflammation, which may support improved CD4^+^ T cell proliferation; however, further research is needed to address this in more detail.

In this study, we demonstrate that an IL-21–based immunomodulatory approach has an adjuvanted effect of improving influenza vaccine–induced antibody responses in SIV^+^ aged RM. Unique to IL-21–treated animals, we observed increased frequencies of TIGIT-expressing pTfh in circulation, which strongly associated with flu vaccine–induced antibody titers, as well as GC Tfh density within LN follicles after B1. IL-21–treated animals had decreased frequencies of CD11b-expressing monocytes, a subset that has been previously identified to be an indicator of systemic inflammation and impaired vaccine-induced antibody responses. Furthermore, an expansion of AM B cells and concomitant contraction of CD21^lo^CD27^–^ B cells in IL-21–treated animals corresponded with increased antibody titers to flu vaccination. The observed alteration of TIGIT^+^ pTfh, AM B cell, and CD11b^+^ monocyte population frequencies in IL-21–treated animals represents immune correlates of influenza vaccine responses in SIV^+^ aged RM and may be indirectly or directly modulated by IL-21 immunotherapy. This is further underscored by sPLS-DA modeling, which selected TIGIT^+^ pTfh, AM/CD21^lo^CD27^–^ B cells, and CD11b expressing monocytes as top variables that best explain immunological differences between IL-21–treated and controls after B1 in our data set. Moreover, Poisson regression modeling demonstrated the strong influence of TIGIT^+^ pTfh, AM B cell, and CD11b^+^ monocyte frequencies and the interactions between them at both baseline (preprime and pre–IL-21 immunotherapy) and after B1 on HAI titers after B1. Poisson modeling of baseline frequencies while controlling for IL-21 immunotherapy revealed that the frequencies of TIGIT^+^ pTfh, AM B cells, and CD11b^+^ monocytes alone and together may represent peripheral biomarkers predictive of future flu vaccine response magnitude. These results further strengthen the evidence for IL-21–mediated immunomodulation, which associates with improved vaccine responses. Given our findings in peripheral blood, we hypothesize that a mechanism of IL-21 immunomodulation may be through lowering CD11b^+^ monocyte frequency alongside expanding TIGIT^+^ pTfh cell frequency, thereby promoting the differentiation of B cells toward AM B cells. While some of the LN alterations associated with aging and protracted HIV infection appear irreversible, such as increase collagen deposition affecting cell-to-cell contact needed for proper immune crosstalk (54), IL-21 appears to be an immunomodulator capable of enhancing antibody responses to flu vaccination in aging and HIV/SIV through altering the Tfh, monocyte, and B cell responses. Further characterization of direct mechanisms by which IL-21 modulates the expansion of TIGIT-expressing CD4^+^ Tcm/pTfh and AM B cells and the suppression of CD11b expression on circulating monocytes is needed.

## Methods

### Animals.

Indian RMs housed at the New Iberia Research Center (NIRC) at the University of Louisiana at Lafayette were used in this study. Prior to study initiation, there was no history of recent/current infection or vaccination. For this study, 16 aged (average: 21 years [y], range: 3.8 y) female (*n* = 15) and male (*n* = 1) and 12 young (average: 4.58 y, range: 6 y) female (*n* = 4) and male (*n* = 8) animals were enrolled, divided into subgroups: young SIV-uninfected IL-21–IgFc–untreated (*n* = 4), aged SIV-uninfected IL-21–IgFc–untreated (*n* = 4), young SIV-infected IL-21–IgFc–untreated (*n* = 4), young SIV-infected IL-21–IgFc–treated (*n* = 4), aged SIV-infected IL-21–IgFc–untreated (*n* = 4), and aged SIV-infected IL-21–IgFc–treated (*n* = 8) as shown in Table 1. For SIV infection, 200 median tissue culture infective dose (TCID_50_) of SIVmac239-*nef-stop* was administered i.v. (37) to avoid using animals with fast SIV progression and allow animals to mount an adaptive immune response to SIV infection (55–59). Infected animals were given ART starting at 12 weeks after infection (early chronic phase), and ART was continued throughout the study period. ART consisted of tenofovir (TFV; 20 mg/Kg/day) combined with emtricitabine (FTC; 30 mg/Kg/day) s.c. (both from Carbosynth) and Raltegravir (L870,812 donated by Merck Sharp & Dohme Corp.) (20 mg/Kg/day up to a maximal dose of 100 mg/day, given orally). Viral loads of SIV infected animals were monitored throughout the course of the study using quantitative PCR (qPCR). All animals were vaccinated intramuscularly at 3 months after ART initiation with the trivalent 2015–2016 seasonal influenza vaccination (Afluria vaccine manufactured by bioCSL carrying 15 μg each of H1N1, H3N2, and B antigens) in a prime/boost/boost strategy at 3-month intervals near regional LNs (inguinal and axillary). In the prime/boost/boost strategy, the rationale for the vaccination regimen of primary vaccination followed by 2 boosts was to determine whether boosting would bring immune responses among aged SIV-infected RMs to levels comparable with aged SIV uninfected. The main objective was to test the adjuvant effect of IL-21 to improve the immune function in a prime-boost vaccination strategy. Three-month intervals were chosen between vaccine doses to allow for immune responses to reach the memory phase between each immunization, similar to humans given 1 dose per year. All SIV^+^ animals were virally suppressed at the time of vaccination and had immune reconstitution comparable with uninfected animals. For IL-21–IgFc treatment, 8 old SIV-infected animals and 4 old SIV-uninfected animals received s.c. IL-21–IgFc (Resource for NHP Immune Reagents), 50 μg/kg body weight in 3 doses: (a) on day –2 before each vaccination at the upcoming vaccination site to prime immune cells, (b) concurrent and colocated to the site of vaccination, and (c) 7 days after vaccination. Blood samples were collected at time points day 0 (before vaccination), day 14, day 42 after each vaccine dose, and as well as day 84 after B2. Blood was processed for PBMC, serum, and plasma for cryopreservation until the time of assay. Draining LN for subsequent in situ analysis were sampled at day 14 after vaccination, fixed (4% PFA), and paraffin embedded. For SIV-infected RM, blood was collected at preinfection and at weekly–biweekly time points before and after initiation of ART to ensure control of viral replication.

### Sample collection and processing.

Collection and processing of blood, rectal biopsies, and LN biopsies were performed as previously described (60–62). Draining LN biopsies were either axillary or inguinal according to the respective site of vaccination. Blood samples were used for a complete blood count and chemical analysis; plasma was separated by centrifugation (room temperature, 400*g*, 10 minutes) within 1 hour of phlebotomy. PBMC were isolated by density gradient centrifugation (room temperature, 400*g*, 30 minutes). Rectal biopsies were performed via anoscope placed a short distance into the rectum, with up to 20 pinch biopsies obtained with forceps. Lymphocytes were isolated from rectal biopsies by digestion with 1 mg/mL collagenase IV from Worthington and RQ1 RNase-free DNAse from Promega for 1 hour at 37°C followed by passage through a 70 mm cell strainer to remove residual tissue. For LN biopsies, the skin over axillary and inguinal regions was clipped and surgically prepared. Incisions were made over the LN, which was exposed via blunt dissection and excised over clamps. LN tissue biopsies were homogenized and passed through a 70 mm cell strainer for mechanical isolation of lymphocytes. Samples were then processed, fixed in 1% paraformaldehyde, and analyzed within 24 hours of collection. A portion of LN biopsies was formalin fixed and paraffin embedded for histological analysis.

### Influenza antibody response.

Influenza virus antibody titers were determined in serum by HAI titers using the 2015–2016 trivalent influenza vaccine performed with serum using chicken RBCs as previously described (12, 63).

### PBMC immunophenotyping.

Cryopreserved cells were thawed and rested overnight at 37°C prior to staining with live/dead aqua (Invitrogen), followed by surface and intracellular staining with titrated concentrations of monoclonal antibodies that cross-react with RMs. Surface-stained cells were permeabilized with BD cytofix/cytoperm solutions and then intracellularly stained. Finally, cells were resuspended in 1% paraformaldehyde, acquired on a BD LSRFortessa instrument, and subsequently analyzed by FlowJo V10 (Tree Star Inc.).

### Monoclonal antibodies.

The following fluorochrome conjugated monoclonal antibodies reactive with macaque cells were used for flow cytometry studies: CD3 (SP34-2, BD Biosciences [BD]), CD4 (L200, BD), CD8 (SK1, BD), CD95 (DX2, BD), CD28 (CD28.2, BioLegend), CCR5 (3A9, BD), CXCR5 (MU5UBEE, Invitrogen), PD-1 (EH12.2H7, BioLegend), ICOS (C398.4A, BioLegend), CCR7 (150503, BD), α4β7 (A4B7, NHP Reagent Resource), LAG3 (3DS223H, eBioscience), Tim-3 (F38-2E2, BioLegend), TIGIT (MBSA43, Invitrogen), CD20 (2H7, BioLegend), CD19 (J3-119, Beckman Coulter), HLA-DR (L243, BioLegend), CD10 (HI10A, BioLegend), CD21 (B-ly4, BD), CD27 (O323, BioLegend), IgD (IADB6, Southern Biotech), IgG (G18-145, BD), IL-21R (2G1-K12, BioLegend), Ki-67 (B56, BD), BCL6 (IG191E/A8, BioLegend), CD80 (2D10, BioLegend), CD56 (B159, BD), CD45 (D058-1283, BD), CD14 (MoP9, BD), CD16 (3G8, BD), CCR2 (48607, R&D Systems), CD11b (ICRF44, BioLegend), CD11c (3.9, Invitrogen), PD-L1 (29E.2A3, BioLegend), PD-L2 (24F.10C12, BioLegend), CD80 (2D10, BioLegend), CD86 (FUN-1, BD), CD141 (1A4, BD), CD163 (GHI/61, BioLegend), and CD123 (7G3, BD).

### T cell flow cytometry panel.

In a panel designed to investigate immune checkpoint markers on CD4^+^ and CD8^+^ T cells and subsets included Live (aqua^–^) CD4^+^ Tcm were defined as CD3^+^CD4^+^CD28^hi^CD95^hi^. pTfh cells were identified as CXCR5^+^ within the CD4^+^ Tcm population. CD4^+^ Tcm and pTfh cells were further characterized by their expression of PD1 and TIGIT.

### B cell flow cytometry panel.

In a panel designed to investigate B cells, subsets were defined as follows: mature B cells (Lin^–^CD20^+^CD10^–^), transitional B cells (Lin^–^CD20^+^CD10^+^), CD27^–^CD10^–^ B cells (Lin^–^CD20^+^CD10^–^CD27^–^), AM B cells (Lin^–^CD20^+^CD10^–^CD21^lo^CD27^+^), resting memory B cells (Lin^–^CD20^+^CD10^–^CD21^hi^CD27^+^), naive B cells (Lin^–^CD20^+^CD10^–^CD21^hi^CD27^–^), and CD21^lo^CD27^–^ B cells were defined as Live (aqua^–^), CD3^–^CD20^+^CD10^–^CD21^lo^CD27^–^.

### Monocyte flow cytometry panel.

In a panel designed to investigate monocytes, total monocytes were inclusive of classical monocytes CD14^+^CD16^–^, inflammatory monocytes CD14^+^CD16^+^, and nonclassical monocytes CD14^–^CD16^+^ and gated from the parent population of Live (aqua^–^), CD45^+^HLADR^+^CD3^–^CD20^–^CD56^–^ cells. For this study, total monocytes were further characterized by their expression of CD86, CD163, and CD11b.

### Tissue staining.

Formalin-fixed paraffin-embedded LN tissues were cut into 5 μm sections, deparaffinized at 60°C, and serially washed in 100% xylene, 95% ethanol, 80% ethanol, and 70% ethanol before 100% diH_2_O baths (Thermo Fisher Scientific). Antigen retrieval was performed for 15 minutes at 110°C in a Borg decloaker (Biocare Medical). Slides were then submerged in 1× PBS for cooling, followed by permeabilization/blocking for 1 hour in PBS (Thermo Fisher Scientific)/bovine albumin (Millipore Sigma)/Triton-X (Thermo Fisher Scientific). Slides were then stained overnight (4°C) with titrated concentrations of primary antibodies. Slides were washed (3 times, 15 minutes each in 1× PBS) and then stained with titrated concentrations of secondary antibodies for 2 hours at room temperature, followed by 3 more washes. Slides were then blocked with mouse and or goat serum for 1 hour, followed by the addition of titrated concentrations of fluorochrome-conjugated antibodies for 1 hour. Slides were then washed 3 times and stained with nuclear stain (JoPro/Thermo Fisher Scientific) for 15 minutes. Coverslips were then mounted using Fluoromont G (Thermo Fisher Scientific). Tissue sections were stained using JoPro, CD3, CD4, PD-1, CD20, Ki-67, and BCL6. Tfh cells were defined as CD3^+^CD4^+^PD1^hi^.

### Tissue imaging.

Tissue images were captured with a Nikon A1 confocal microscope operated by NIS-Elements AR software. Images were acquired using a 20× (NA 0.75) dry lens and 40× (NA 1.3) oil lens. NIS-Elements AR software was used to stitch multiple fields of view and *Z* stacks. Each field of view had a pixel density of 512 × 512. No frame averaging or summing was utilized during image capture. Emitted fluorescence was deconvoluted using “live unmixing” in NIS-Elements AR, utilizing singe-stained tissues from an emission spectrum database to calculate the contribution of the known individual fluorophore’s spectra to the total collected signal. Histocytometry analysis was performed as previously reported (64).

### PCA and sPLS-DA modeling.

To verify the observed immunomodulatory effects of IL-21 on day 14 after B1, we employed sPLS-DA. sPLS-DA is a supervised clustering machine-learning algorithm for multivariate dimensionality reduction, variable selection via lasso penalization, and classification (41). Prior to running sPLS-DA, we employed unsupervised PCA as an indicator of sPLS-DA reliability on our flow cytometry data set (41). Because PCA modeling is unsupervised, without input of group identity, a combined 53% explained variance from PC1 and PC2 — in addition to spatial separation of IL-21–treated versus –untreated animals when plotting PC1 against PC2 — supports the reliability of sPLS-DA as a tool for both variable selection and classification in our data set (41).

Optimal sPLS-DA model tuning and performance was determined by assessing the balanced classification error rate. We found the optimal parameters (number of components and number of selected phenotypes for each component) for our sPLS-DA model by implementing a 4-fold cross-validation repeated 50 times to predict class membership (aged SIV^+^IL-21^+^ versus aged SIV^+^IL-21^–^). The PCA and sPLS-DA analyses were implemented using the mixOmics package in R (R Foundation for Statistical Computing) (65).

### Poisson regression modeling.

We employed a top-down exploratory model selection approach to build 2 separate Poisson regression models in order to determine the effects of the frequency of 3 phenotypes (TIGIT^+^ pTfh, AM B cell, and CD11b^+^ Total Monocytes) at baseline and at 14 days after boost one on Influenza titer level at 14 days post-boost one, while also controlling for IL-21 immunotherapy. Poisson regression modeling was performed using R.

### Statistics.

Cross sectional flow cytometry and histocytometric-derived data were analyzed using 2-tailed Mann-Whitney *U* tests. Longitudinal flow cytometry data comparisons were analyzed using 2-way ANOVA with Tukey’s multiple-comparison test. Between-group comparisons of longitudinal flow cytometry were analyzed using 2-way ANOVA with Sidak’s multiple-comparison test. Longitudinal HAI titer data were analyzed by mixed-effects model with Fisher’s least-significant difference (LSD) post hoc multiple-comparison correction. Correlations between flow cytometry data and HAI titers were performed using Spearman correlation. One-tailed spearman correlations were performed for evidence-based directional *a priori* hypothesis testing (correlations between HAI titers and TIGIT^+^ pTfh, AM B cells, and CD11b^+^ monocytes). All other spearman correlations were 2-tailed. A *P* value less than 0.05 was considered significant. All data shown are displayed with mean ± SEM. All statistical analysis shown was generated using GraphPad Prism 8.4.1.

### Study approvals.

All animals were maintained in accordance with the Animal Welfare Act and the NIH guidelines for housing and care of laboratory animals, and experiments were performed in accordance with institutional regulations after review and approval by the IACUC of the University of Louisiana at Lafayette.

## Author contributions

DK and TS performed experiments, data analysis, and initial draft writing; S. Pallikkuth performed experimental design, data analysis, and manuscript revision; AK performed multivariate data analysis and initial draft writing; RP performed data analysis and manuscript revision; CP performed experimental design, tissue analysis, and manuscript revision; FV and SP provided intellectual input, study conception, experimental design, funding acquisition, and final draft revision. Order of equally contributing authors was decided based on their contributions.

## Supplementary Material

Supplemental data

## Figures and Tables

**Table 1:**
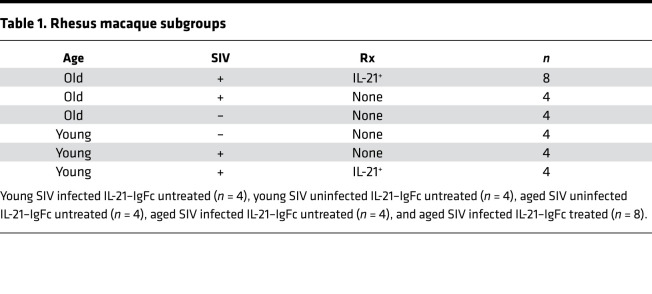
Rhesus macaque subgroups

**Table 2:**
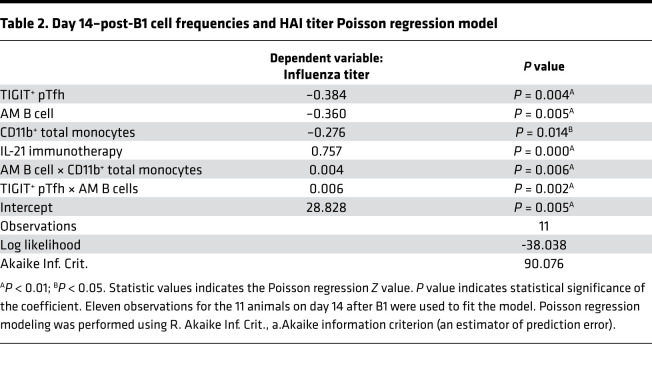
Day 14–post-B1 cell frequencies and HAI titer Poisson regression model

**Table 3:**
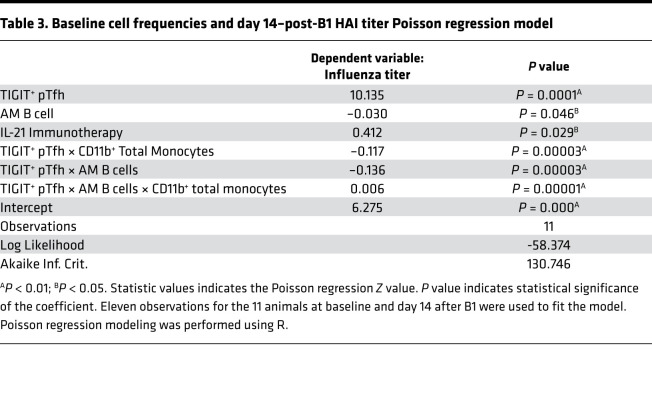
Baseline cell frequencies and day 14–post-B1 HAI titer Poisson regression model

## Version 1 09/07/2021

In-Press Preview

## Version 2 10/22/2021

Electronic publication
