# Supplementary material for: IL-21 enhances influenza vaccine responses in aged macaques with suppressed SIV infection
Source: JCI Insight. 2021 Oct 22;6(20):e150888. doi: 10.1172/jci.insight.150888 (PMC8564910; doi:10.1172/jci.insight.150888)
Supplement: Supplemental data [file jciinsight-6-150888-s210.pdf]

Supplementary Figures

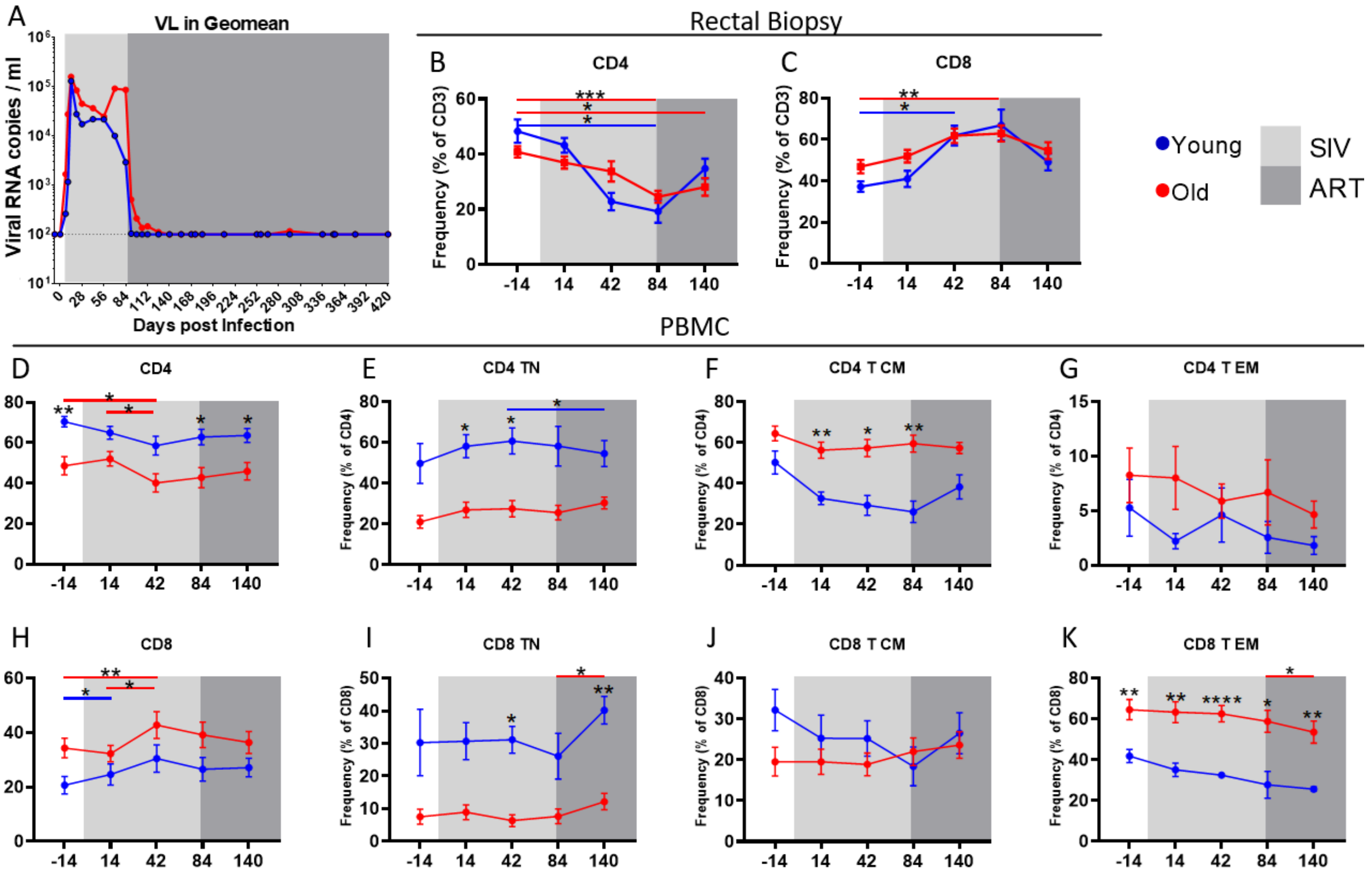

Figure S1. Rhesus macaque model of aging and SIV infection. (**A**) Geometric mean of the plasma viral load (VL) in young (n=4) and old (n=12) infected animals. (**B**) longitudinal CD4 and CD8 T cell frequencies of CD3+ LIVE/Dead- cells isolated from rectal biopsies in young (n=4) and old (n=12) animals. (**C**) longitudinal peripheral blood mononuclear cell (PBMC) CD4 and CD8 frequencies of CD3+ LIVE/Dead- cells as well as CD4 and CD8 memory subset frequencies including T naïve (TN), TCM (T central memory) and effector memory (EM) in young (n=4) and old (n=12) animals. Data is displayed as mean ± SEM, * *p* <0.05, ** *p* <0.005, *** *p* <0.005, Longitudinal comparisons and young vs old comparisons were performed with two-way ANOVA using Fisher's LSD post hoc multiple comparisons correction.

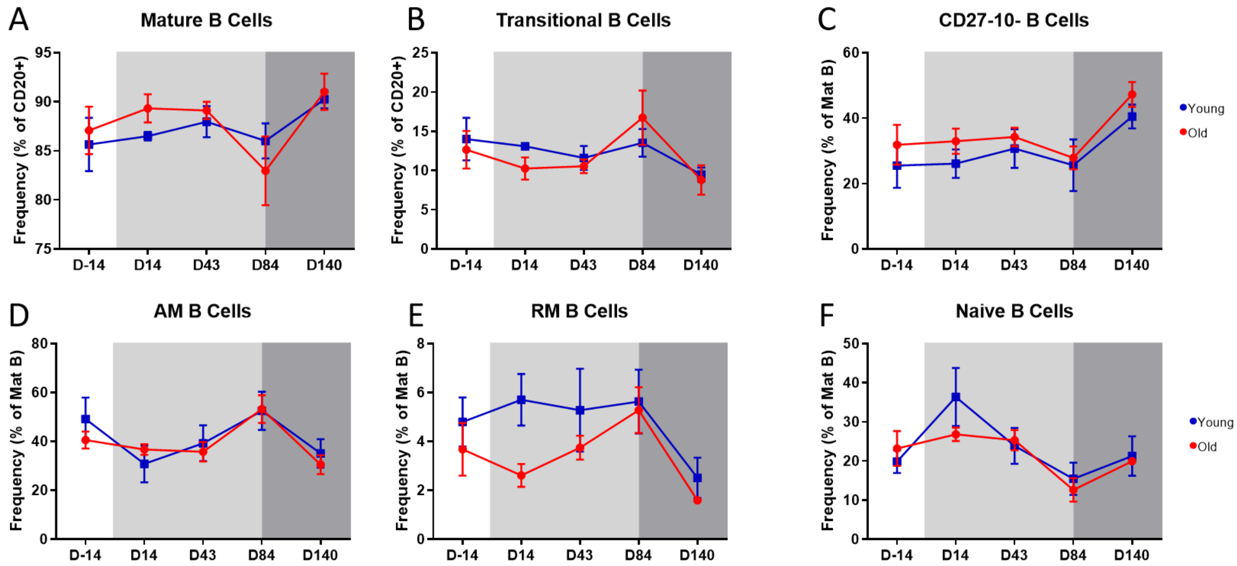

Figure S2: Rhesus macaque model of aging and SIV infection: SIV and ART phase PBMC B cell subset frequencies. (**A**) Mature B cells (Lin-CD20+CD10-), and (**B**) Transitional B cells (Lin-CD20+CD10+), are shown as frequency of CD20+ cells. (**C**) CD27-CD10- B cells (Lin-CD20+CD10-CD27-), (**D**) Activated memory B cells (Lin-CD20+CD10-CD21loCD27+), (**E**) resting memory B cells (Lin-CD20+CD10-CD21hiCD27+) and (**F**) naïve B cells (Lin-CD20+CD10-CD21hiCD27-) are shown as frequency of mature B cells for young (n=4) and old (n=12) animals. Light grey shading indicates time of SIV infection, dark grey shading indicates ART therapy start and duration. Longitudinal comparisons and young vs old comparisons were performed with two-way ANOVA using Fisher's LSD post hoc multiple comparisons correction.

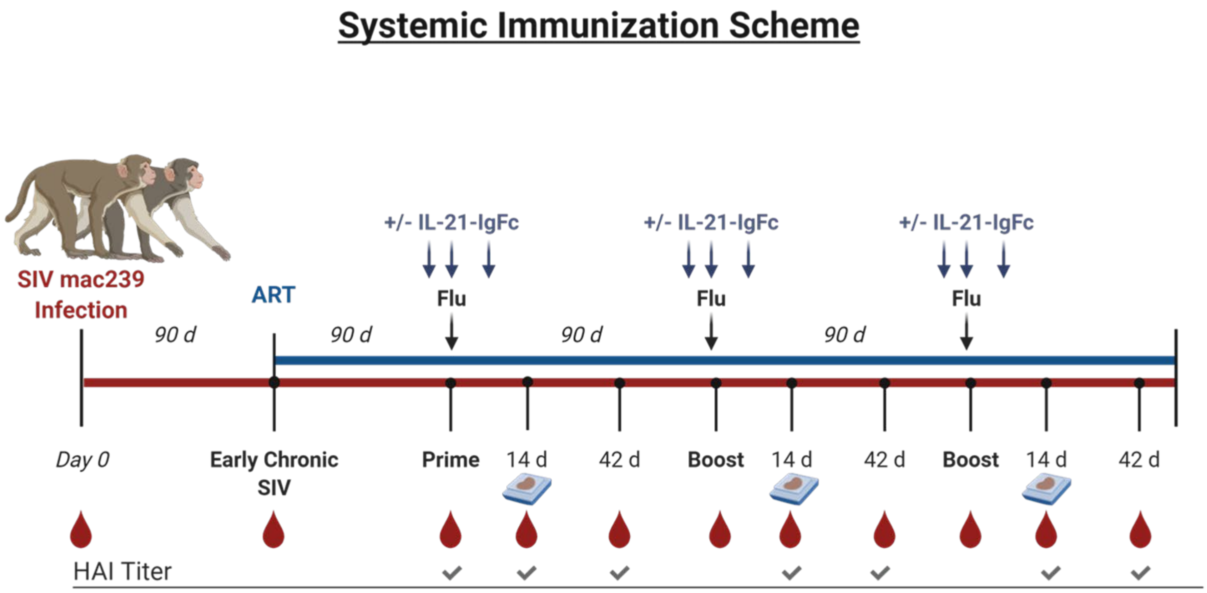

Figure S3. Schematic illustration of protocol for SIV infection, ART treatment, systemic influenza immunization and sample collection. SIVmac239 was administered at 200 TCID50, IV. ART was initiated 90 days post infection PMPA/FTC/L-000870812. 90 days post ART-initiation the trivalent 2015-2016 seasonal influenza vaccine (Afluria vaccine manufactured by bioCSL with 15 µg each of H1N1, H3N2 and B antigens) was administered intramuscularly in a prime-boost-boost strategy at 3-month intervals. IL-21-IgFc [50µg/kg body weight] was administered subcutaneously in 3 doses: 1) on day -2 before each vaccination at the upcoming vaccination site to prime immune cells, 2) concurrent and co-located to the site of vaccination, and 3) 7 days post vaccination. Red blood spot cartoons indicate days of blood collection for PBMC, plasma and serum isolation; draining LN collection on D14 post vaccine timepoints are indicated by cartoon of formalin fixed paraffin embedded block.

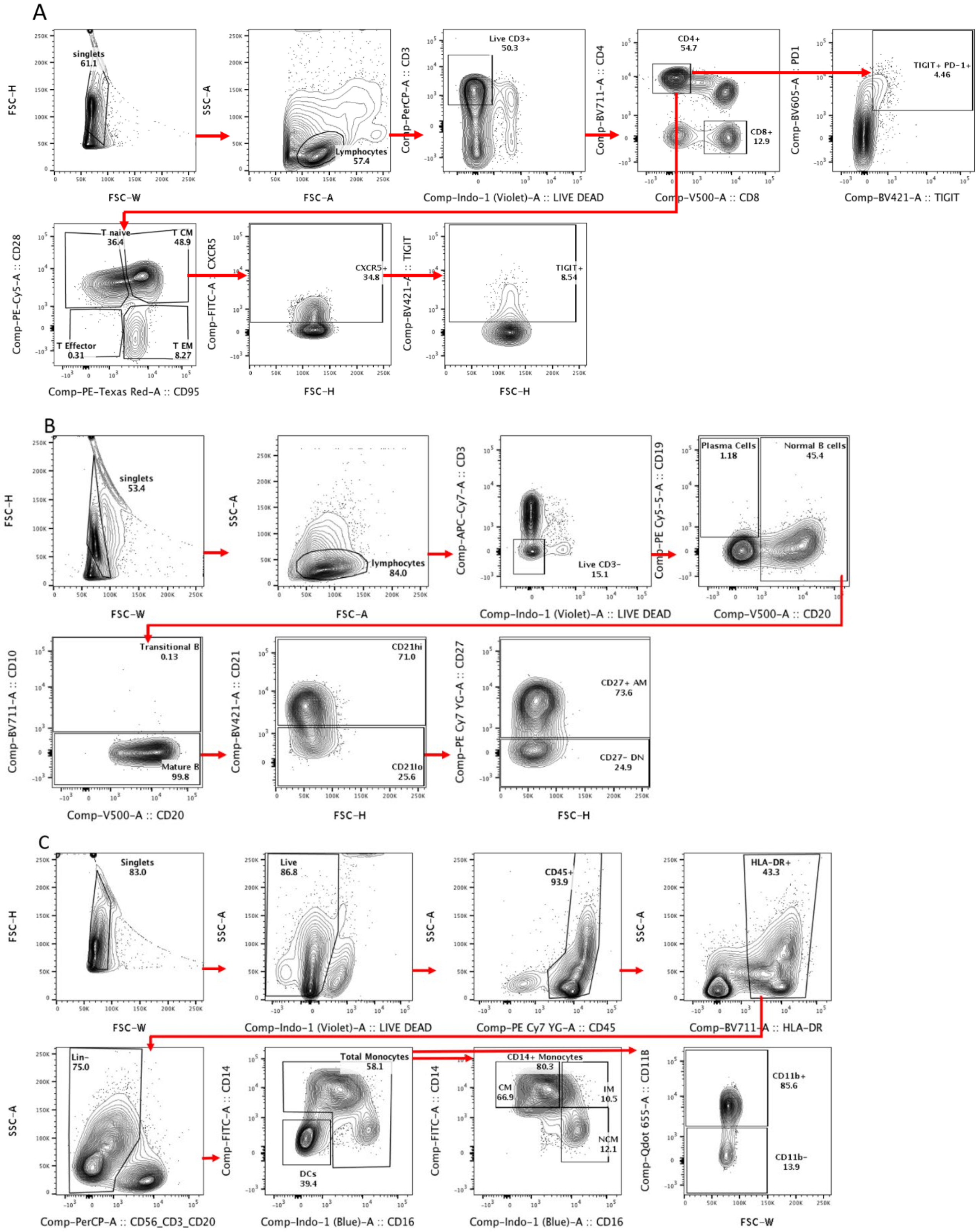
A
singlets 61.1
FSC-H
FSC-W
SSC-A
Lymphocytes 57.4
FSC-A
Comp-PerCP-A :: CD3
Live CD3+ 50.3
Comp-Indo-1 (Violet)-A :: LIVE DEAD
Comp-BV711-A :: CD4
CD4+ 54.7
CD8+ 12.9
Comp-V500-A :: CD8
Comp-BV605-A :: PD1
TIGIT+ PD-1+ 4.46
Comp-BV421-A :: TIGIT
Comp-PE-Cy5-A :: CD28
T naive 36.4
T CM 48.9
T Effector 0.31
T EM 8.27
Comp-PE-Texas Red-A :: CD95
Comp-FITC-A :: CXCR5
CXCR5+ 34.8
FSC-H
Comp-BV421-A :: TIGIT
TIGIT+ 8.54
FSC-H
B
singlets 53.4
FSC-H
FSC-W
SSC-A
lymphocytes 84.0
FSC-A
Comp-APC-Cy7-A :: CD3
Live CD3- 15.1
Comp-Indo-1 (Violet)-A :: LIVE DEAD
Comp-PE Cy5-5-A :: CD19
Plasma Cells 1.18
Normal B cells 45.4
Comp-V500-A :: CD20
Comp-BV711-A :: CD10
Transitional B 0.13
Mature B 99.8
Comp-V500-A :: CD20
Comp-BV421-A :: CD21
CD21hi 71.0
CD21lo 25.6
FSC-H
Comp-PE Cy7 YG-A :: CD27
CD27+ AM 73.6
CD27- DN 24.9
FSC-H
C
Singlets 83.0
FSC-H
FSC-W
SSC-A
Live 86.8
Comp-Indo-1 (Violet)-A :: LIVE DEAD
SSC-A
CD45+ 93.9
Comp-PE Cy7 YG-A :: CD45
SSC-A
HLA-DR+ 43.3
Comp-BV711-A :: HLA-DR
SSC-A
Lin- 75.0
Comp-PerCP-A :: CD56_CD3_CD20
Comp-FITC-A :: CD14
Total Monocytes 58.1
DCs 39.4
Comp-Indo-1 (Blue)-A :: CD16
Comp-FITC-A :: CD14
CD14+ Monocytes 80.3
CM 66.9
IM 10.5
NCM 12.1
Comp-Indo-1 (Blue)-A :: CD16
Comp-Qdot 655-A :: CD11B
CD11b+ 85.6
CD11b- 13.9
FSC-W

Figure S4: Flow cytometry gating strategies (**A**) Representative figures showing gating strategy used for CD4+ and CD8+ T cells, maturation subsets, pTfh (CD4+/T CM/CXCR5+) and gating for TIGIT+ and TIGIT+PD-1+ populations. (**B**) Representative figures showing gating strategy used for normal B (CD20+), transitional B (CD20+CD10+), mature B (CD20+CD10-) and CD20+CD10-CD21loCD27+ activated memory and CD20+CD10-CD21loCD27- double negative B cell populations. (**C**) Representative figures showing gating strategy used for monocyte subsets. Total monocytes were considered CD45+HLA-DR+Lin- and inclusive of classical monocytes (CD14+CD16-), intermediate monocytes (CD14+CD16+) and non-classical monocytes (CD14-CD16+). Gating for total monocyte CD11b+ population is also shown. Analysis was performed with FlowJo (Treestar).

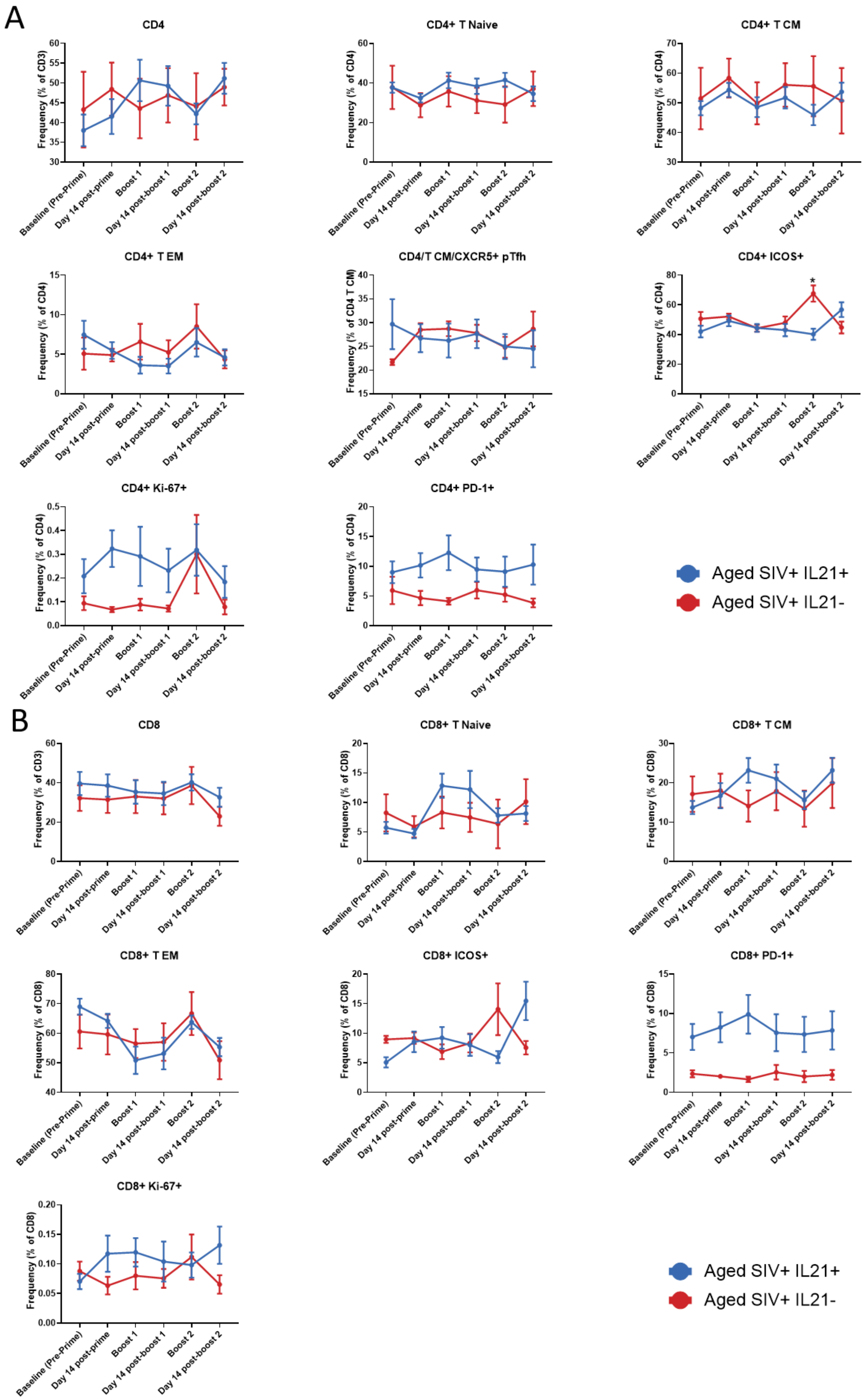
A
CD4
CD4+ T Naive
CD4+ T CM
CD4+ T EM
CD4/T CM/CXCR5+ pTfh
CD4+ ICOS+
CD4+ Ki-67+
CD4+ PD-1+
Frequency (% of CD3)
Frequency (% of CD4)
Frequency (% of CD4 T CM)
Baseline (Pre-Prime)
Day 14 post-prime
Boost 1
Day 14 post-boost 1
Boost 2
Day 14 post-boost 2
Aged SIV+ IL21+
Aged SIV+ IL21-
B
CD8
CD8+ T Naive
CD8+ T CM
CD8+ T EM
CD8+ ICOS+
CD8+ PD-1+
CD8+ Ki-67+
Frequency (% of CD8)
Aged SIV+ IL21+
Aged SIV+ IL21-

Figure S5: Longitudinal vaccine phase Immune phenotyping. Longitudinal frequencies of total (**A**) CD4 and (**B**) CD8 as well as respective maturation subset distributions and expression of markers including ICOS, Ki-67 and PD-1 for aged SIV+ IL21+ (n=8), and aged SIV+ IL21- (n=4). * *p* <0.05, Longitudinal comparisons and young vs old comparisons were performed with two-way ANOVA using Fisher's LSD post hoc multiple comparisons correction.

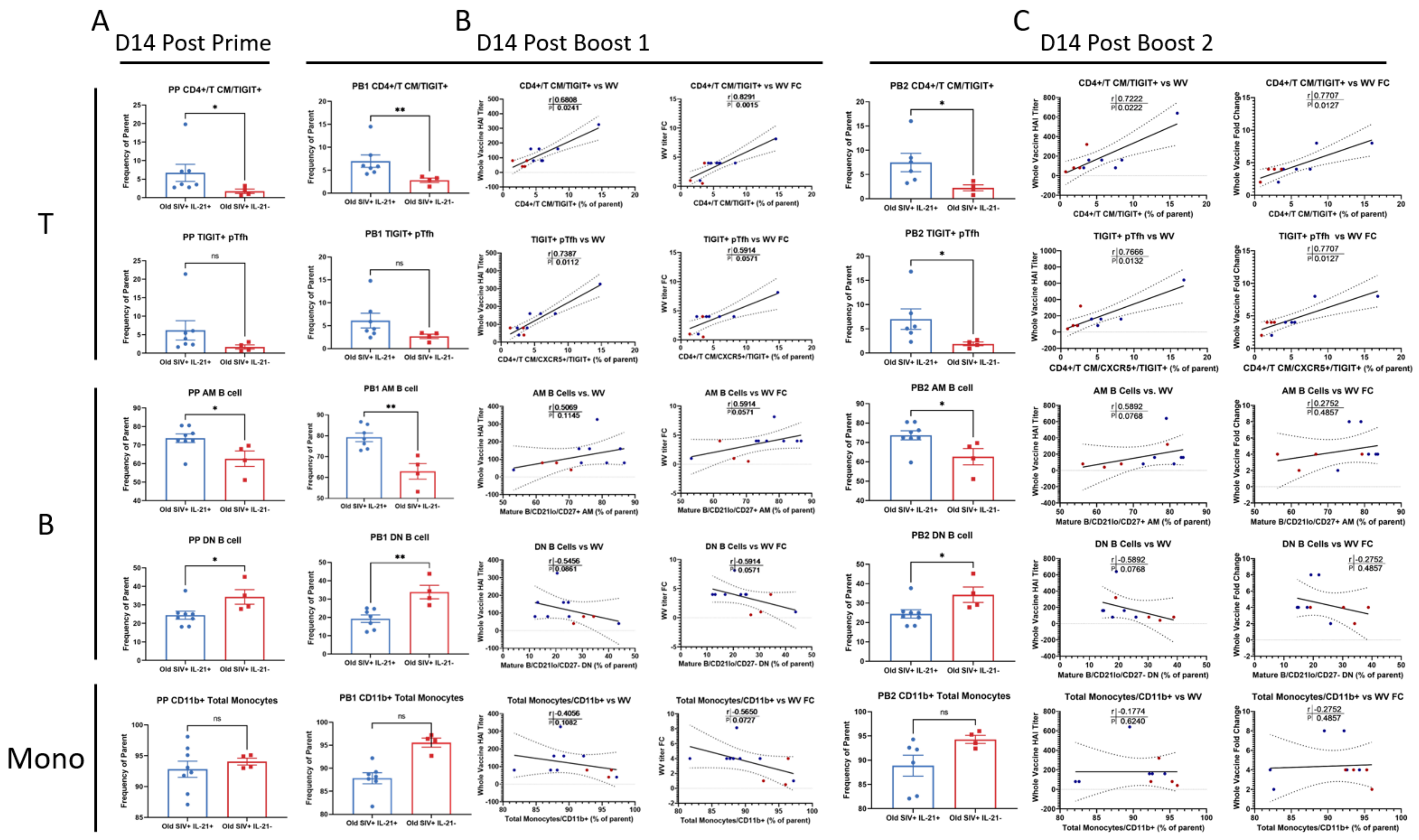
A
D14 Post Prime
B
D14 Post Boost 1
C
D14 Post Boost 2
T
B
Mono
PP CD4+/T CM/TIGIT+
PP TIGIT+ pTfh
PP AM B cell
PP DN B cell
PP CD11b+ Total Monocytes
PB1 CD4+/T CM/TIGIT+
PB1 TIGIT+ pTfh
PB1 AM B cell
PB1 DN B cell
PB1 CD11b+ Total Monocytes
PB2 CD4+/T CM/TIGIT+
PB2 TIGIT+ pTfh
PB2 AM B cell
PB2 DN B cell
PB2 CD11b+ Total Monocytes
Frequency of Parent
Old SIV+ IL-21+
Old SIV+ IL-21-
CD4+/T CM/TIGIT+ vs WV
r 0.6808
P 0.0241
CD4+/T CM/TIGIT+ vs WV FC
r 0.8291
P 0.0015
TIGIT+ pTfh vs WV
r 0.7387
P 0.0112
TIGIT+ pTfh vs WV FC
r 0.5914
P 0.0571
AM B Cells vs. WV
r 0.5069
P 0.1145
AM B Cells vs WV FC
r 0.5914
P 0.0571
DN B Cells vs WV
r -0.5456
P 0.0861
DN B Cells vs WV FC
r -0.5914
P 0.0571
Total Monocytes/CD11b+ vs WV
r -0.4056
P 0.1082
Total Monocytes/CD11b+ vs WV FC
r -0.5650
P 0.0727
CD4+/T CM/TIGIT+ vs WV
r 0.7222
P 0.0222
CD4+/T CM/TIGIT+ vs WV FC
r 0.7707
P 0.0127
TIGIT+ pTfh vs WV
r 0.7666
P 0.0132
TIGIT+ pTfh vs WV FC
r 0.7707
P 0.0127
AM B Cells vs. WV
r 0.5892
P 0.0768
AM B Cells vs WV FC
r 0.2752
P 0.4857
DN B Cells vs WV
r -0.5892
P 0.0768
DN B Cells vs WV FC
r -0.2752
P 0.4857
Total Monocytes/CD11b+ vs WV
r -0.1774
P 0.6240
Total Monocytes/CD11b+ vs WV FC
r -0.2752
P 0.4857
Whole Vaccine HAI Titer
WV titer FC
Whole Vaccine Fold Change
CD4+/T CM/TIGIT+ (% of parent)
CD4+/T CM/CXCR5+/TIGIT+ (% of parent)
Mature B/CD21lo/CD27+ AM (% of parent)
Mature B/CD21lo/CD27- DN (% of parent)
Total Monocytes/CD11b+ (% of parent)

Figure S6: D14 post-prime, post-B1 and post-B2 Immune phenotypes and correlation with HAI titer. (**A**) D14 post-prime (PP) peripheral TIGIT+ frequencies of CD4+ TCM, pTfh (CD4+/TCM/CXCR5+), D14 post-prime peripheral AM and CD21loCD27- (DN) B cell frequencies, as well as D14 post-prime peripheral CD11b+ total monocyte frequency. (**B**) D14 post-B1 (PB1) peripheral TIGIT+ frequencies of CD4+ TCM, pTfh (CD4+/TCM/CXCR5+), D14 post-B1 peripheral AM and CD21loCD27- (DN) B cell frequencies, as well as D14 post-B1 peripheral CD11b+ total monocyte frequency, and correlations between subset frequencies and D14 post-B1 whole vaccine HAI titers and HAI titer fold change from day of B1. (**C**) D14 post-B2 (PB2) peripheral TIGIT+ frequencies of CD4+ TCM, pTfh (CD4+/TCM/CXCR5+), D14 post-B2 peripheral AM and CD21loCD27- (DN) B cell frequencies, as well as D14 post-B2 peripheral CD11b+ total monocyte frequency, and correlations between subset frequencies and D14 post-B2 whole vaccine HAI titers and HAI titer fold change from day of B2. * $p < 0.05$, Mann Whitney test and Spearman R correlations performed.

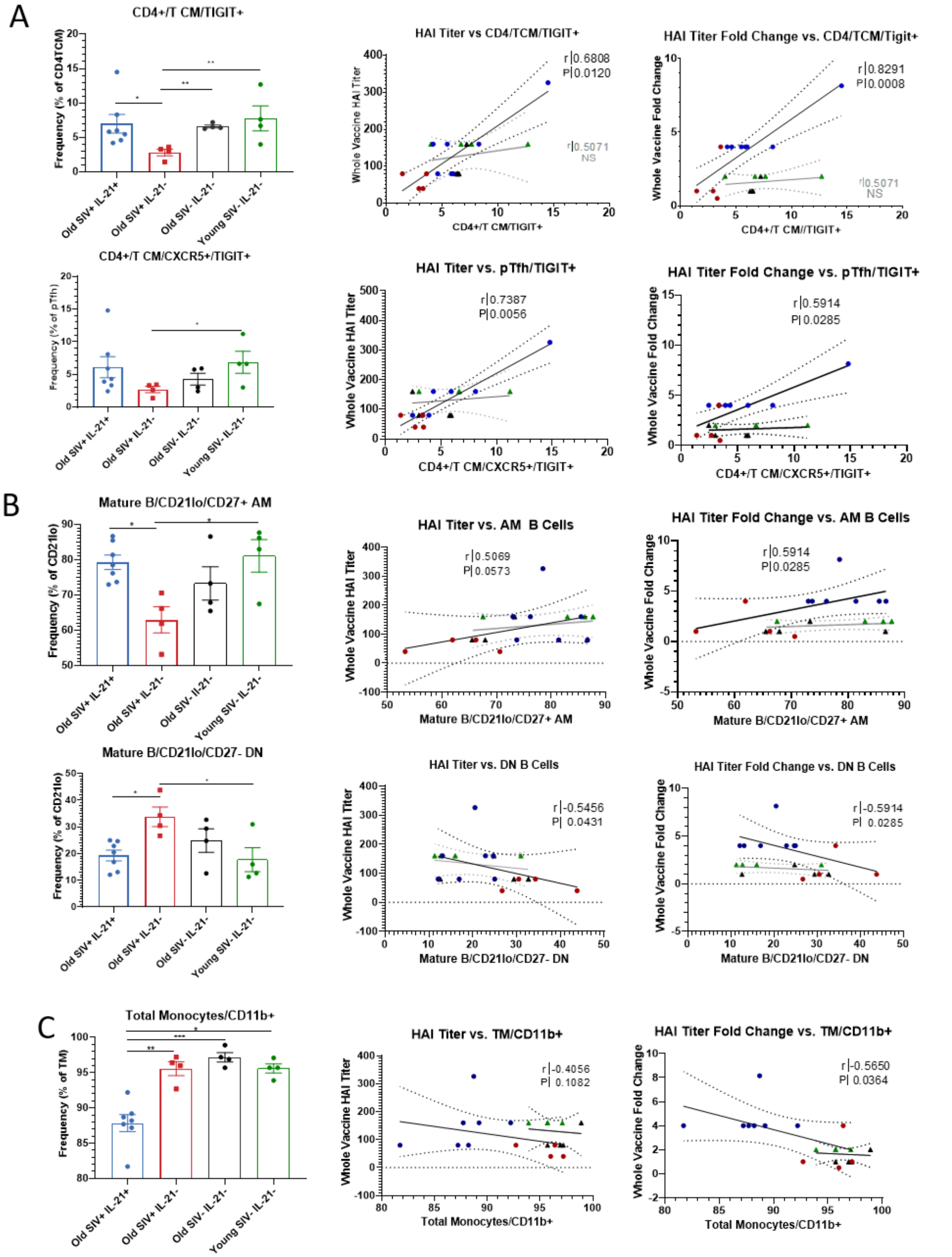

Figure S7. <u>Post-B1 peripheral T, B and monocyte subsets and correlation with whole vaccine HAI titers including SIV- IL-21-untreated old and young controls</u> (**A**) D14 post-B1 TIGIT+ frequencies of CD4+ TCM and pTfh (CD4+/TCM/CXCR5+) and correlations with D14 post-B1 whole vaccine HAI titers and HAI titer fold change from boost 1 baseline. Blue dots represent old SIV+ IL-21+ animals (n=8), red dots represent old SIV+ IL-21- animals (n=4) black dots represent old SIV- IL-21- (n=4) and green dots represent young SIV- IL-21- animals (n=4) (**B**) D14 post-B1 CD27+ AM B cell and CD27- DN B cell frequencies of CD21lo mature B cells and correlations with D14 post-B1 whole vaccine HAI titers and HAI titer fold change from boost 1 baseline. (**C**) D14 post-B1 CD11b+ frequencies of total monocytes (including Lineage-, CD14+CD16-, CD14+CD16+, and CD14-CD16+) and correlation with D14 post-B1 whole vaccine HAI titers and HAI titer fold change from boost 1 baseline. * p <0.05, ** p <0.005, *** p <0.0005 Mann Whitney test and Spearman R correlations performed.

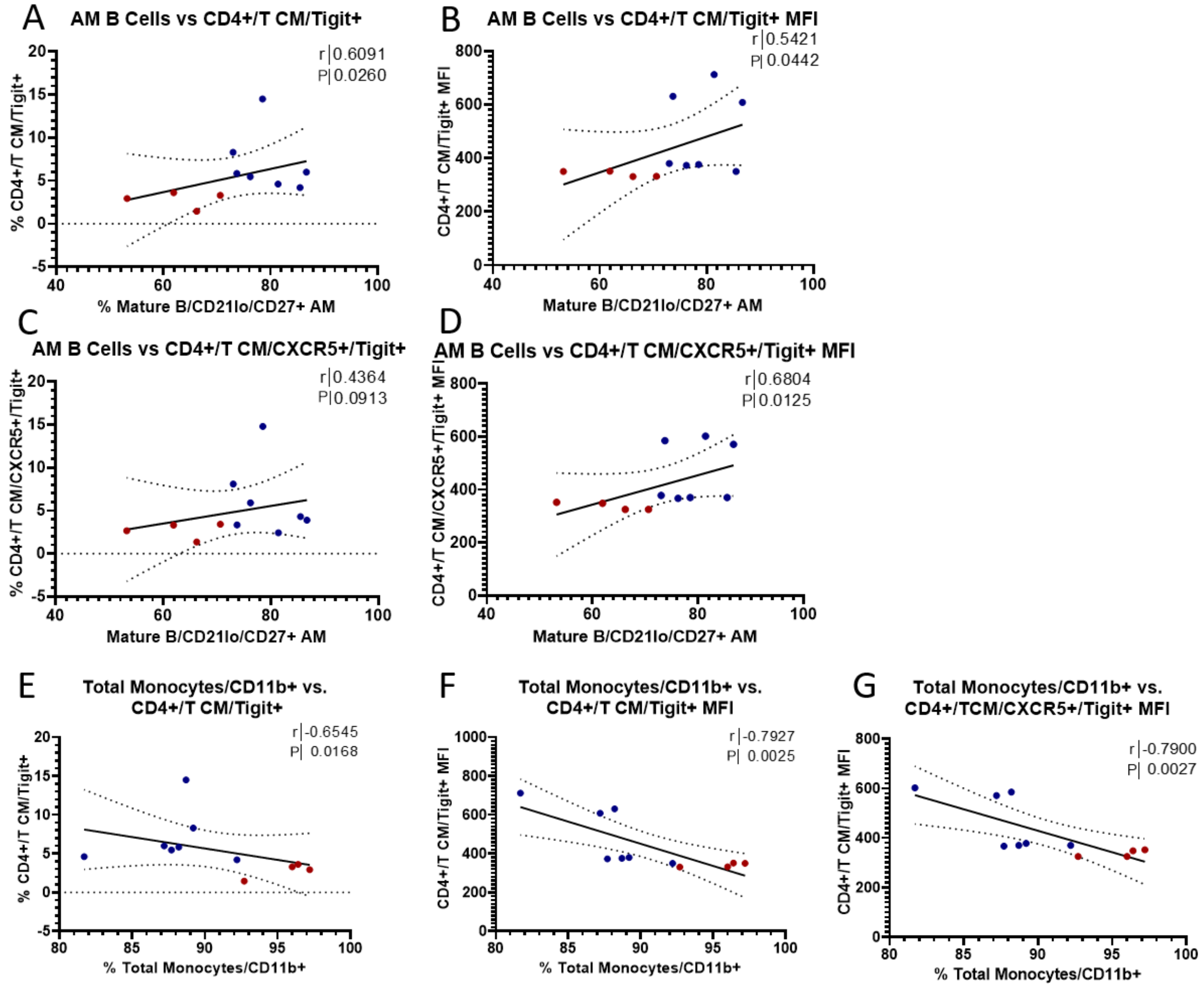

Figure S8. Post-B1 PBMC Immune subset Interrelationships (**A**) inter-subset correlations between D14 post-B1 cell subset frequencies/MFI. Blue dots represent old SIV+ IL-21+ animals (n=8), while red dots represent old SIV+ IL-21- animals (n=4). Spearman R correlations were performed.

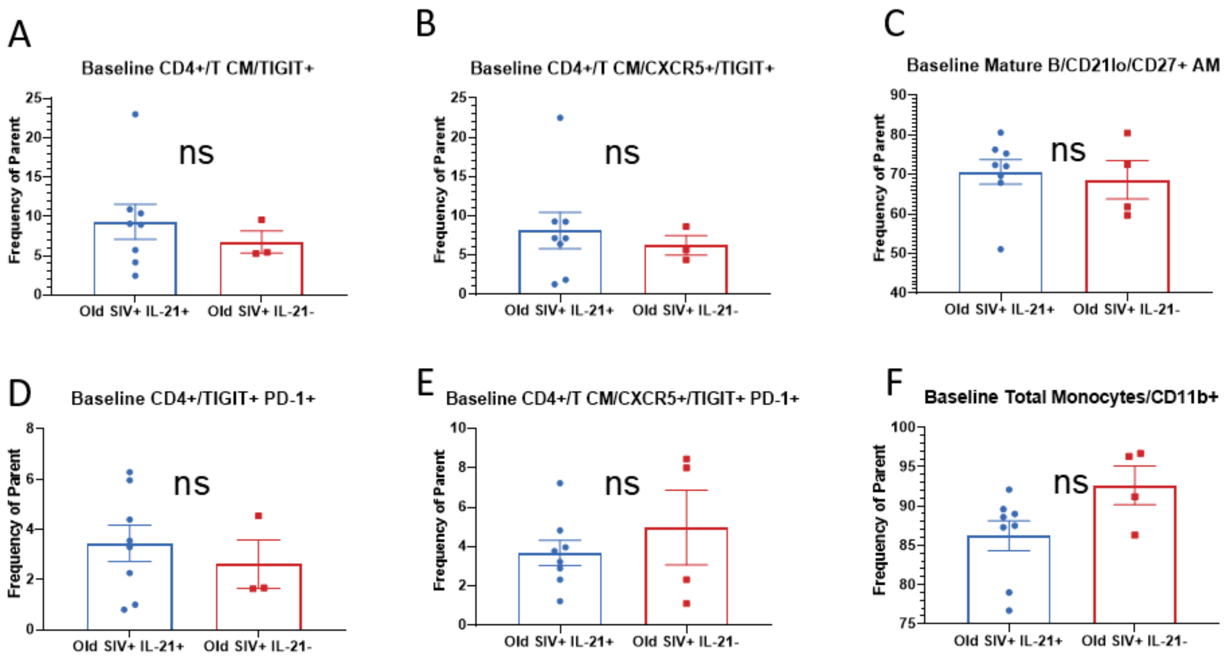

Figure S9: Pre-vaccination baseline frequencies of (**A**) CD4+/TCM/TIGIT+, (**B**) (CD4+/TCM/CXCR5+/TIGIT+), (**C**) CD27+ AM B cell frequencies of CD21lo mature B cells, (**D**) TIGIT+ PD-1+ double positive frequencies of total CD4+ and (**E**) TIGIT+ PD-1+ double positive CD4+ TCM, (**F**) as well as baseline frequencies of CD11b+ total monocytes. Mann Whitney tests were performed.

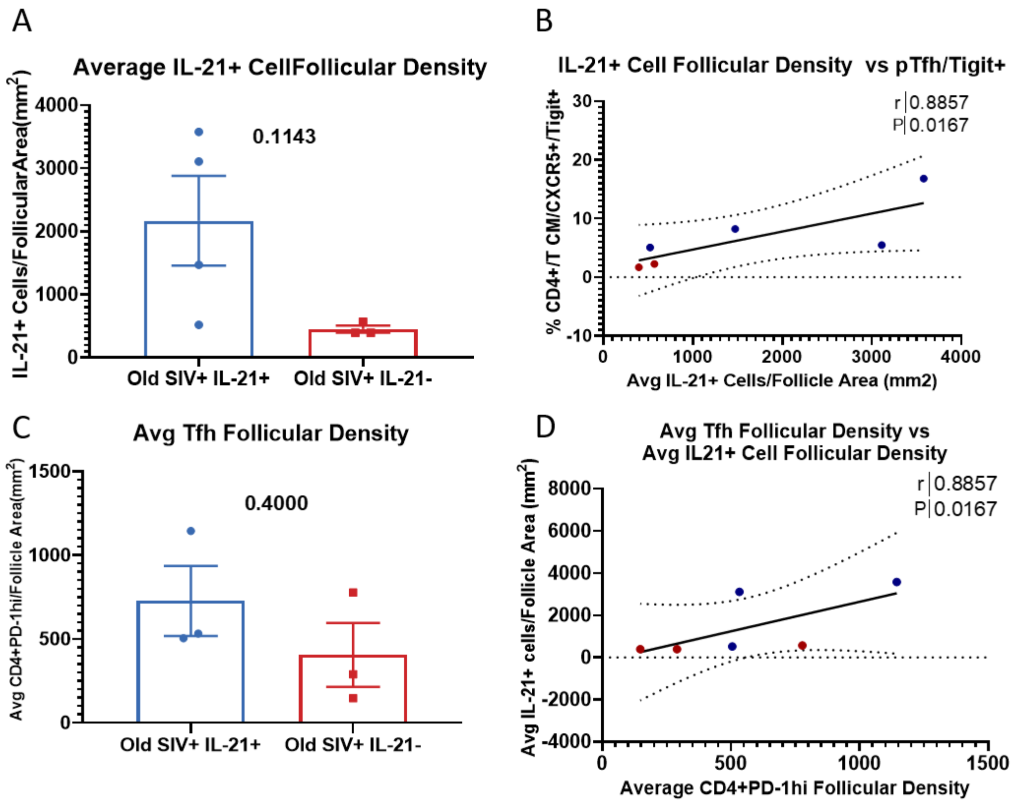

Figure S10: D14 post-B2 LN follicle cell densities and correlations. (**A**) D14 post-B2 LN tissue IL-21+ and (**C**) Tfh CD4+PD-1hi average cell densities/follicle. (**B**) Correlation between peripheral D14 post-B2 TIGIT+ pTfh (CD4+/TCM/CXCR5+) and D14 post-B2 average LN follicle IL-21+ cell density. (**D**) Correlation between D14 post-B2 average LN follicle IL-21+ cell density and average LN follicle density of Tfh (CD4+PD-1+) cells. Mann Whitney test and Spearman R correlations were performed.

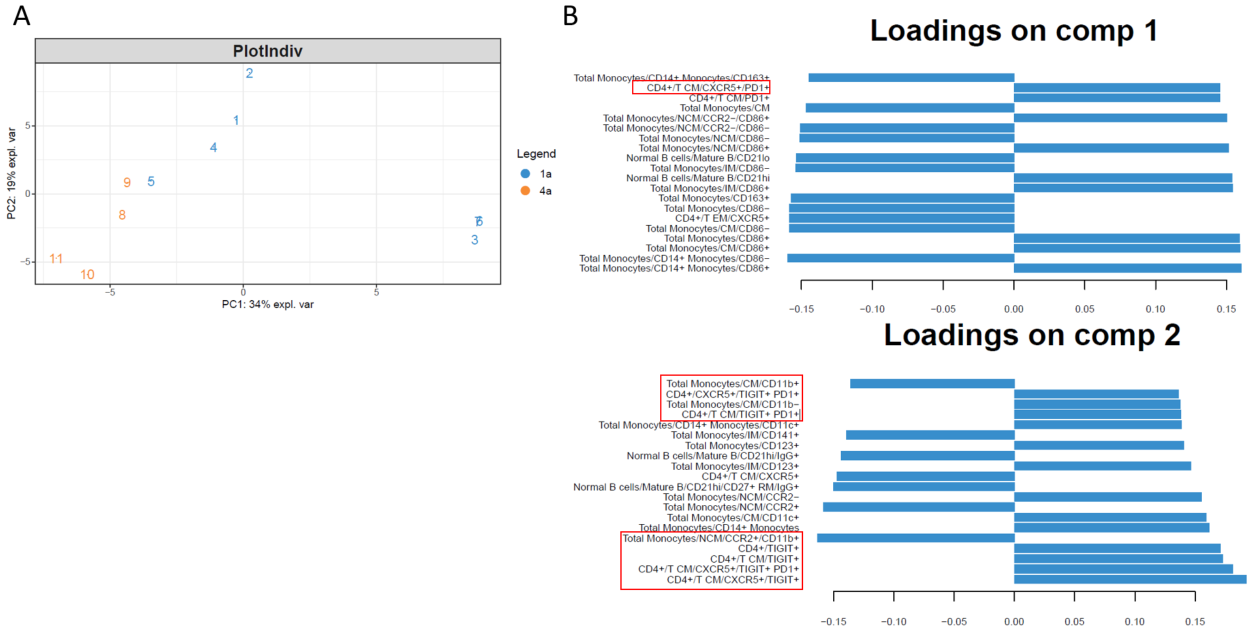

Figure S11. Unsupervised PCA analysis reveals separation of IL-21 treated vs untreated animals and indicates favorable sPLS-DA reliability.

(A) PCA components 1 and 2 plotted on x and y axis respectively. Plotted numbers indicate individual animals, with blue indicated IL-21 treated animals (n=7) and orange indicating controls (n=4). (B) The top 20 loading variables and their loading scores are shown for PCA components 1 and 2. Populations similar to those identified to be altered by IL-21 immunotherapy through manual univariate analysis are indicated by red boxes.
